# Of Travertine and Time: Otolith Chemistry and Microstructure Detect Provenance and Demography of Endangered Humpback Chub in Grand Canyon, USA

**DOI:** 10.1371/journal.pone.0084235

**Published:** 2013-12-16

**Authors:** Karin E. Limburg, Todd A. Hayden, William E. Pine, Michael D. Yard, Reinhard Kozdon, John W. Valley

**Affiliations:** 1 Department of Environmental and Forest Biology, State University of New York College of Environmental Science and Forestry, Syracuse, New York, United States of America; 2 Great Lakes Fishery Commission, Ann Arbor, Michigan, United States of America; 3 Department of Wildlife Ecology and Conservation, University of Florida, Gainesville, Florida, United States of America; 4 United States Geological Survey, Grand Canyon Monitoring and Research Center, Southwest Biological Science Center, Flagstaff, Arizona, United States of America; 5 Department of Geoscience, University of Wisconsin-Madison, Madison, Wisconsin, United States of America; North Carolina State University, United States of America

## Abstract

We developed a geochemical atlas of the Colorado River in Grand Canyon and in its tributary, the Little Colorado River, and used it to identify provenance and habitat use by Federally Endangered humpback chub, *Gila cypha*.  Carbon stable isotope ratios (δ^13^C) discriminate best between the two rivers, but fine scale analysis in otoliths requires rare, expensive instrumentation. We therefore correlated other tracers (SrSr, Ba, and Se in ratio to Ca) to δ^13^C that are easier to quantify in otoliths with other microchemical techniques. Although the Little Colorado River’s water chemistry varies with major storm events, at base flow or near base flow (conditions occurring 84% of the time in our study) its chemistry differs sufficiently from the mainstem to discriminate one from the other. Additionally, when fish egress from the natal Little Colorado River to the mainstem, they encounter cold water which causes the otolith daily growth increments to decrease in size markedly. Combining otolith growth increment analysis and microchemistry permitted estimation of size and age at first egress; size at first birthday was also estimated. Emigrants < 1 year old averaged 51.2 ± 4.4 (SE) days and 35.5 ± 3.6 mm at egress; older fish that had recruited to the population averaged 100 ± 7.8 days old and 51.0 ± 2.2 mm at egress, suggesting that larger, older emigrants recruit better. Back-calculated size at age 1 was unimodal and large (78.2 ± 3.3 mm) in Little Colorado caught fish but was bimodally distributed in Colorado mainstem caught fish (49.9 ± 3.6 and 79 ± 4.9 mm) suggesting that humpback chub can also rear in the mainstem. The study demonstrates the coupled usage of the two rivers by this fish and highlights the need to consider both rivers when making management decisions for humpback chub recovery.

## Introduction

It is a lo[a]thesome little stream, so filthy and muddy that it fairly stinks. It is only 30 to 50 [yards] wide now and in many places a man can cross it on the rocks without going on to his knees ... [The Little Colorado was] as disgusting a stream as there is on the continent ... half of its volume and 2/3 of its weight is mud and silt. [It was little but] slime and salt ... a miserably lonely place indeed, with no signs of life but lizards, bats and scorpions. It seemed like the first gates of hell. One almost expected to see *Cerberus poke* his ugly head out of some dismal hole and growl his disapproval of all who had not Charon's pass.

• *George* *Bradley and Jack* *Sumner, August 1869* [1, p. 234]

The Colorado River of the southwestern US and northwestern Mexico supports critical ecosystem and human services to seven western US states, numerous federally recognized tribes, and several Mexican states. The Colorado River provides water to more than 40 million people, substantial hydroelectric production, and billions of dollars in agricultural products [2]. One of the most striking features of this river basin is Grand Canyon, a UNESCO World Heritage site of cultural, geological, and biological significance protected by the Grand Canyon Protection Act of 1992. President Theodore Roosevelt declared that this is “…the one great site every American should see” and more than 4 million people follow this advice annually to visit Grand Canyon National Park.

 The Colorado River in Grand Canyon is an approximately 400-km canyon bound river reach between Lakes Powell and Mead, the two largest reservoirs in the US. Lake Powell, created following the completion of Glen Canyon Dam in 1963, is effectively the primary source of the Colorado River in Grand Canyon and the dam regulates the timing, duration, and magnitude of river flows to meet hydropower demands and downstream water release obligations. Lake Powell also retains most of the sediment from the upper Colorado River basin, and both reservoir depth and volume as well as river discharge control water temperatures throughout most of Grand Canyon [3,4]. In the post-Glen Canyon Dam environment, the Colorado River currently transports about <10% of the historic sediment load (current inputs are from tributaries [5]) and water temperatures are cool (8°C to 10°C from 1994-2002 [6]) with minimal seasonal variation. This is in stark contrast with its undammed state, a strongly seasonally fluctuating environment.

The Colorado River basin historically supported more than 40 endemic fish species and 8 of these species were historically found in Grand Canyon. Of these species, four are thought extinct in Grand Canyon (roundtail chub *Gila robusta*, bonytail chub *G. elegans*, Colorado pikeminnow *Ptychocheilus lucius*, and razorback sucker *Xyrauchen texanus*). Of the remaining fish species, the largest known population of humpback chub *Gila cypha*, a large-bodied, US federally listed (Endangered) cyprinid, is found in nine “aggregations” throughout Grand Canyon, including the largest aggregation near the confluence of the Colorado and Little Colorado rivers. This “Little Colorado River aggregation” likely accounts for more than 90% of the humpback chub in Grand Canyon [7] and the Little Colorado River provides the largest known spawning and rearing habitat for humpback chub in Grand Canyon. Current understanding of humpback chub life history suggests that adults undertake a potamodromous spawning migration to the unregulated Little Colorado River in the spring, after which they return to the mainstem Colorado River [7-9]. Later in the season, juvenile humpback chub may either (1) emigrate to the mainstem Colorado River as larvae and small juveniles where their survival was thought to be low because of low post-dam water temperatures [10] and predation from non-native species [11] or (2) rear in the Little Colorado River for 1-3 years where growth rates were higher due to warm water and predation risk lower before joining the adult spawning migration [8,9,12]. 

Based on long-term mark-recapture sampling, adult humpback chub populations in Grand Canyon declined through the 1990’s and early 2000’s [7,9]. The reasons for this decline are unknown [13] but the leading theories include (1) predation by or competition with non-native fish [11,14], (2) loss of required habitats following completion of Glen Canyon Dam related to changes in river flow and sediment supply [15,16], (3) cold, hypolimnetic water releases from Glen Canyon Dam [17,10]. These population declines motivated experimental management actions in Grand Canyon including non-native fish removal [14], translocations to tributary systems to minimize extinction risk [18], and experimental floods to re-build sandbar habitats to stop population declines and aid in population recovery [19].

The Little Colorado River is the largest tributary of the Colorado River in Grand Canyon; is spring fed in the segment of interest (lower 21 km); and receives spring runoff from snowmelt as well as precipitation, typically in summer monsoons. Base flows in the perennially flowing lower 21 km of the Little Colorado River are maintained by springs including Blue Spring, a major upwelling from deep groundwater that brings up carbonated water from a karst aquifer [20]. Upon contact with air, the groundwater de-gasses, causing supersaturated calcium and magnesium to precipitate as carbonate in travertine (Figure S1 in File S1) [20-22] among other reactions. As a result, carbon isotopes fractionate as the dissolved inorganic carbon (DIC) in the flowing water equilibrates with the atmosphere and mineralization occurs [22]. This travertine accumulates naturally to create dams in the Little Colorado River that act as barriers to fish migration, restricting humpback chub primarily to the lower 14 km of the Little Colorado River below the large travertine dam that creates Chute Falls [8,16]. Little Colorado River flows vary seasonally, with larger flows occurring during periods of spring runoff and summer monsoon rains. 

**Figure 1:**
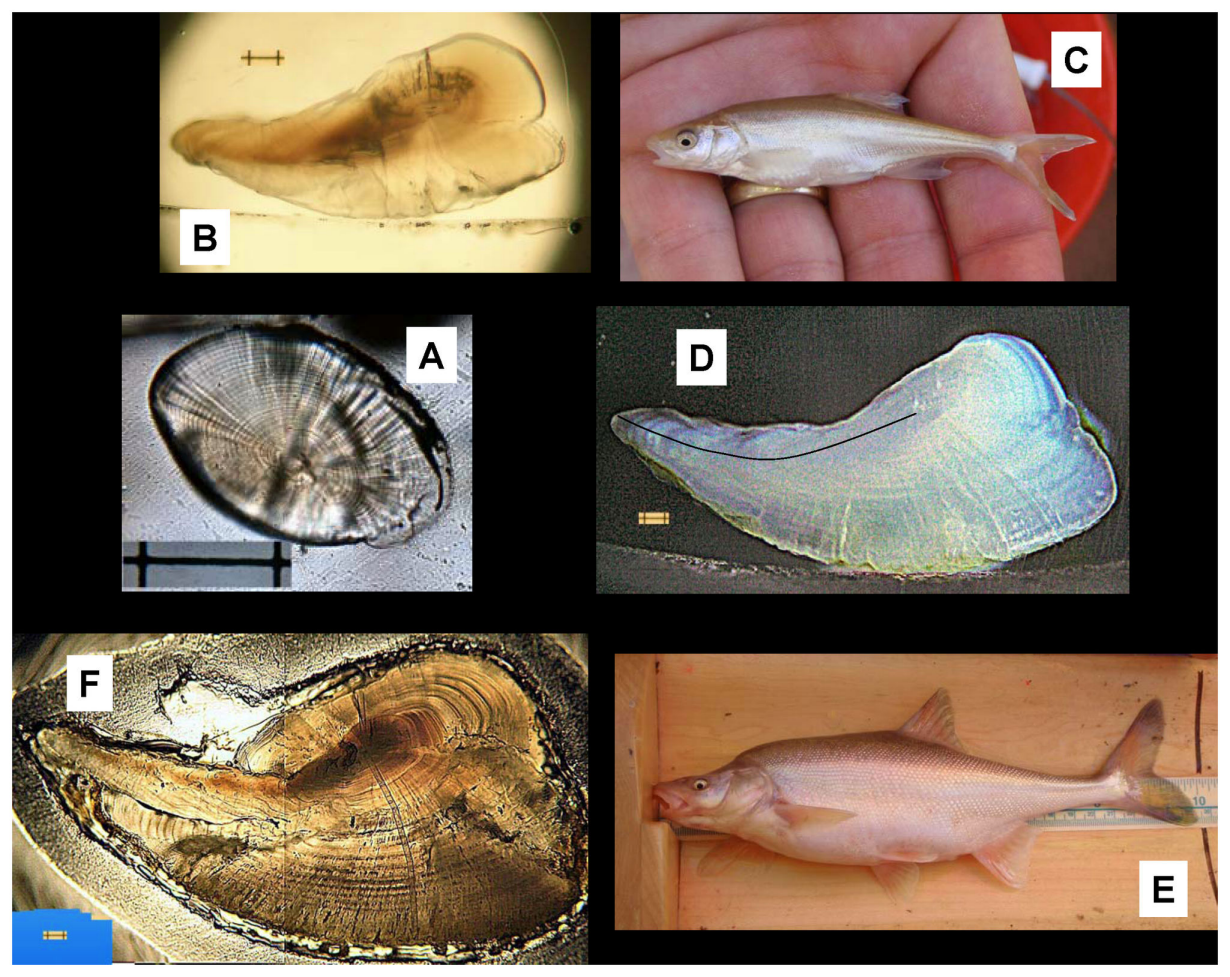
Collage of humpback chub and their lapillar otoliths. Older, larger fish have larger, more curved otoliths. Clockwise from left center: A – otolith of a 20 mm post-larva with 32 daily growth increments; B – otolith from a 2 year old, 114-mm subadult; C – subadult humpback chub (Photo: C. Finch); D – otolith from a 7 year old, 230-mm adult, with line drawn along the axis of measurement; E – old humpback chub (Photo: C. Finch); F – otolith with laser ablation track from a 28 year old, 383-mm chub. Bar on otolith images = 100 microns.

 Previously we presented preliminary data [23] showing that carbon isotopic ratios (expressed as δ^13^C) in otoliths (earstones) of humpback chub matched δ^13^C of end-member waters (the Little Colorado River and the mainstem Colorado River). Briefly, otoliths are calcified structures that form part of the hearing and balance system in teleost (modern) fishes, residing in the inner ear canals. These accrete daily by precipitating aragonite (CaCO_3_) on a protein matrix, laying down growth bands similar to tree rings (Figure 1). For this reason, otoliths are routinely used to assess age and growth in fishes, and play an essential role in fisheries stock assessments [25].

Increasingly, the chemical constituents of otoliths are studied to infer environmental histories of fishes [e.g., 24], and when combined with age and growth information, provide powerful means of interpreting fish life histories in spatially explicit contexts [e.g., 23, 25]. Essentially, each individual fish carries its own “black box recorder” encoding age, growth, and environmental conditions experienced by the fish. A wealth of techniques have emerged for micro-scale chemical analysis, such that trace elemental and isotopic analyses are now feasible where once they were not [24].

Here, we develop a more in-depth study of the relationship of otolith chemistry to water chemistry in this system. We then combine otolith chemistry with age and growth information to elucidate humpback chub spatial demography in this region and attempt to address questions related to the provenance and residence of humpback chub in this reach of the Colorado River. This is important because controversial, and expensive, management actions are directed at improving habitat conditions for humpback chub in the mainstem Colorado River, but not in the Little Colorado. Specifically, we hypothesize that humpback chub that immigrate as small young-of-the-year (YOY) into the Colorado River are less likely to survive and recruit to the subadult/adult population. Conversely, humpback chub that remain longer in the natal tributary habitat (Little Colorado River), which is warmer and more productive, should grow better and therefore recruit to the adult population more successfully. Such findings might then lend weight to policy reform to expand the “recognized ecosystem boundaries” for humpback chub to include the Little Colorado River. 

## Materials and Methods

### Ethics Statement

Collection of all water and fish samples was approved by the two entities responsible for managing this land. One is the National Park Service, part of the US Federal Government.  These are public lands that are managed by the Park Service.  Some of the water samples came from land that is part of the Navajo Nation and is managed by Navajo Nation Department of Fish and Wildlife, who also permitted our work. 

Otolith extraction can only be humanely done by sacrificing fish, as the structures are located in the skull beneath the braincase. Because of their Endangered status under the US Endangered Species Act [26], directed sampling of large numbers of humpback chub for otolith analyses was not possible. Our samples instead came primarily from incidental mortalities that occurred as part of standard fish monitoring activities by cooperating fisheries research agencies or (for adult humpback chub) fish found dead from unknown causes.  A few fish (n < 15 juveniles less than 4 months old) were sacrificed by lethal overdose of anesthetic. Collecting permits for fish and water were obtained from Arizona Game & Fish Department (Scientific Collecting Permit SP790940); U.S. Fish and Wildlife Service Federal Fish and Wildlife Permit (TE212896-0); Navajo Nation Department of Fish and Wildlife (Scientific Collecting Permit 586); and the U.S. National Park Service (Scientific Research and Collecting Permit GRCA-2011-SC1-0041).  Animals were handled in accordance with animal welfare protocols at the University of Florida (IFAS ARC Permit 001-09FAS). All fish samples are in are in possession of the U.S. Geological Survey’s Grand Canyon Monitoring and Research Center (GCMRC), and were made available to us as a loan for extracting the otoliths, after which the bodies were returned to the official repository at the GCMRC.

### Water collection

Field campaigns were conducted from May 2009 - September 2012. Regular sampling was conducted monthly from July - October, 2009 - 2011, with special collections in May 2009, June 2010, and September 2012. Water samples for trace elemental analyses were allowed to settle so that sediments were removed; then samples were decanted into clean bottles and acidified. Samples for stable isotope analysis were filtered (45 μm GF/F) into clean 125-mL bottles, care being taken to leave no head space. All samples were stored, cool, in the dark until returned from the field. 

### Fish collection

**Figure 2:**
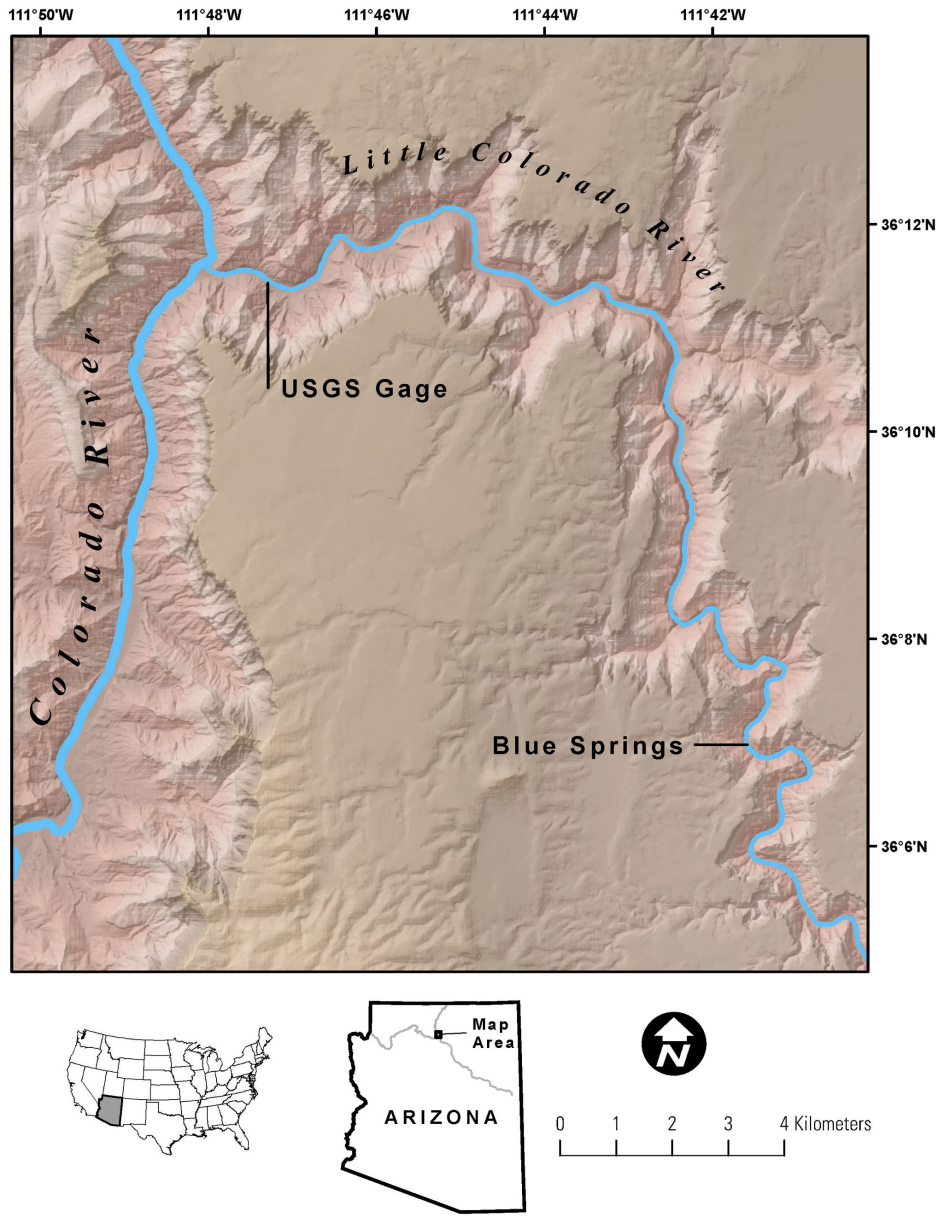
Map of study area, showing Little Colorado River from its confluence with the mainstem of the Colorado River up to Blue Spring, the source of elevated δ^13^C.

Fish samples came from the Little Colorado and mainstem Colorado Rivers downstream of the Little Colorado River between river kilometers (rkm) 102-106 (as measured from Lees Ferry, AZ) between 1995-2012 with 4 adults available from 1995-2000 and the majority of fish (n = 119) based on sampling since 2000 (Figure 2). The majority of samples were collected during May-October 2009-2011 in both the Little Colorado and mainstem Colorado rivers by sampling for 10-12 days each month for 4 or 5 months each year. In the Little Colorado River, fish collections were made using un-baited hoop nets (50-cm diameter x 100-cm length, 10-cm throat, 6-mm nylon mesh); as in [16] where they were generally fished for 24 hours before being checked for fish captures. In the mainstem Colorado River, similar hoop nets were used and in addition slow-speed boat electrofishing (pulsed DC current, 15-20 amps, 200-300 volts, boat speed 7-10 seconds per meter of shoreline, repeated 24 to 72 hours apart for 3 to 5 total passes per trip) was also conducted at night. Electrofishing is not possible in the Little Colorado River due to naturally high conductivity.

 Lapillar otoliths were used, as they form the clearest increments in cypriniform fishes; in contrast, the sagittal otoliths are thin and brittle. Lapilli were dissected from the fish, cleaned, dried, and embedded in epoxy (EpoFix or Epoxicure). Once embedded, frontal plane sections were cut with an Isomet diamond saw (Buehler), polished down to 3 μm with successively finer lapping paper (3M). Otoliths that were prepared for secondary ion mass spectrometry (SIMS) were embedded separately in circular (2.5 cm i.d.) molds, each with 2 pieces of calcite standard (UWC-3 [27]), and polished down to < 1 μm using a powered polishing wheel fitted with diamond-infused polishing cloths. Samples prepared for scanning X-ray fluorescence microscopy (SXFM) were glued to fused quartz glass with cyanoacrylite glue (Loctite). All samples were cleaned by ultrasonication prior to analysis.

### Water chemistry analyses

Bulk elemental analyses were conducted at the SUNY College of Environmental Science and Forestry Analytical and Technical Services laboratory. Selenium, lead, and zinc were analyzed with inductively coupled plasma mass spectrometry (ICPMS, PerkinElmer Elan DRC-e) in aqueous mode. The remaining major, minor, and trace elements (Ca, Ba, Cu, Fe, K, Mg, Mn, Rb, and SrSr) were analyzed via inductively coupled plasma optical emission spectrometry (PerkinElmer Optima 3300DV). Standards were analyzed every 10^th^ analysis; samples that failed QA/QC (> 10% RSD (coefficient of variation)) were re-run until they passed. Only Ca, Ba, Se, and SrSr are reported on here.

 Water samples were also analyzed for carbon stable isotopic ratios. These were sent to the University of California Davis Stable Isotope Facility where δ^13^C_DIC_ was analyzed with a Surveyor HPLC coupled to a ThermoFinnigan Delta Plus Advantage isotope ratio mass spectrometer (Thermo Scientific, Bremen, Germany) through a liquid chromatography Isolink interface [28]. Isotopes in dissolved organic carbon (δ^13^C_DOC_) were analyzed with an O.I. Analytical Model 1030 TOC Analyzer (OI Analytical, College Station, TX) interfaced to a PDZ Europa 20-20 isotope ratio mass spectrometer (Sercon Ltd., Cheshire, UK) utilizing a GD-100 Gas Trap Interface (Graden Instruments). To obtain a weighted average of the δ^13^C_DIC+DOC_, δ^13^C values were multiplied by the respective concentrations of DIC and DOC and summed (Table S1 in File S1).

### Otolith age and growth analysis

Otoliths were photographed with transmitted light microscopy (40-630X) for measurement and age determination. ImageJ [29] was used to enhance growth bands and make measurements. Annual growth bands (annuli) were counted on optical images or sometimes directly at the microscope; elemental maps (see next section) were used as supplementary information to help locate annuli. Similarly, daily increments were counted from digital images and were often double-checked at the microscope. Daily increment formation in lapilli was validated by Hendrickson [67]. Each otolith was read at least twice. In fish that were inferred to have migrated (as evidenced by a change in otolith chemistry and growth increment width, see below), daily increments were enumerated from the core region (laid down around time of hatch) to the point where the growth bands were too narrow to distinguish, indicating very slow growth. Often such slow growth coincided with a change in otolith chemistry [23].

 An otolith length (OL) – fish total length (TL) relationship was developed by measuring the anterior axis of growth, which is the longest one (Figure 1D). The axis becomes curved in age, so a relationship was fit by nonlinear estimation. The best fit was obtained with the following equation: 

$$TL=e^{{\left(\frac{OL}{2.7079}\right)}^{0.267088}}$$ (1)

**Figure 3:**
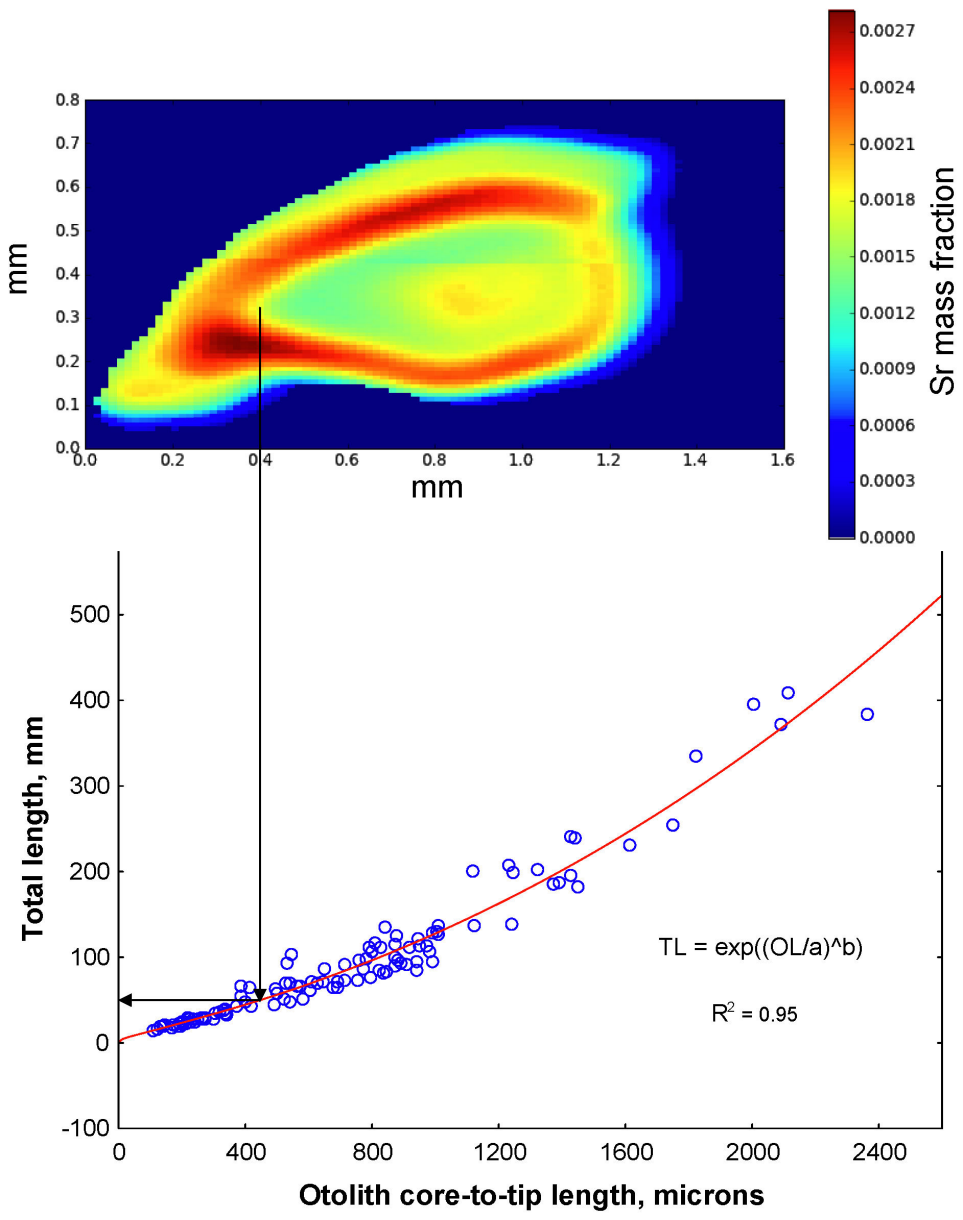
Illustration of back-calculation of size at first egress. Top figure is a strontium map of a humpback chub otolith; below it is the total length-otolith length nonlinear fit. The distance on the longest growth axis (anterior) to a migration event (elevated SrSr) is measured; the corresponding total length is computed using Equation 1.

R^2^ = 0.95. This equation was able to fit the expected values through the data points of the smallest individuals (Figure 3). 

 This equation was used in tandem with otolith chemistry to back calculate fish TL at egress from the Little Colorado River to the Colorado mainstem and corresponding age at egress. A migration event detected in the otolith chemistry (Figure 3) and increment spacing was measured on the otolith and the corresponding size was computed. Total length at formation of the first annulus (corresponding to first “birthday”) was also back-calculated in similar fashion. In this case, a bimodal size distribution was found, and the peaks were quantified with the R package “mixdist” (Ichthus Systems [30]). 

### Otolith chemistry analyses

Ninety-seven otoliths were analyzed with any of three methods for this study, and some were analyzed with more than one method for cross-validation (Table 1). Sixty-nine otoliths were analyzed with scanning X-ray fluorescence microscopy (SXFM) at the Cornell University High Energy Synchrotron Source (CHESS) as described in [31,32,23]. Briefly, trace elements were quantified by stepping a focused beam (10 μm x 20 μm) across the specimen surface with a photon flux of approximately 10^11^ counts per second. Element-specific fluorescences were counted with a Vortex energy-dispersive silicon drift detector fitted with an aluminum foil attenuator to reduce high-intensity calcium fluorescence and increase sensitivity to trace elements. Calibrations were made using an in-house standard made of finely powdered otoliths pressed into a pellet [32]. Two-dimensional maps of each element of interest were produced by rastering each point for 3 seconds. Data reduction and processing were completed using PyMCA [33] and in-house software developed at CHESS to produce the maps and spatially explicit numerical output (mass fraction). Maps (matrices) of Se, Sr, and occasionally other trace elements were divided by the corresponding Ca map to produce maps of trace element:Ca ratios. 

**Table 1:** Numbers of individual fish used for different otolith chemistry analyses.

**SXFM**	**SIMS**	**LA-ICPMS**	**N**
X			52
	X		5
		X	20
X		X	9
X	X		4
	X	X	3
X	X	X	4
		**Total**	97

In situ carbon stable isotope analyses were performed using a CAMECA ims-1280 ion microprobe at the WiscSIMS Laboratory (Wisconsin Secondary Ion Mass Spectrometer), UW-Madison. A ^133^Cs^+^ primary ion beam with an intensity of ~600 pA was focused to a diameter of ~8 µm. The typical ^12^C^¯^ ion intensity was 7 x 10^6^ cps, and ^12^C and ^13^C ions were simultaneously collected by a Faraday Cup detector and an electron multiplier, respectively. Each analysis took ~6 minutes. During each session, 4-6 analyses of the UWC-3 standard (δ^13^C = -0.91‰ [PDB], [27]) were made before and after each set of 6 - 11 sample measurements. The average spot-to-spot reproducibility or external precision of each set of bracketing standards is ±0.78‰ (2 S.D.). The gain of the electron multiplier was monitored before the third analysis of each group of four standard calcite analyses, and the applied high voltage was adjusted to compensate drift of the gain of the electron multiplier, if necessary. Reproducibility of the δ^13^C of the bracketing standard analyses includes the drift of the gain of the electron multiplier. A detailed description of the analytical conditions and the instrument setup for carbon isotope measurement has been published previously [34]. 

A total of 388 δ^13^C measurements in 16 otoliths were performed, including 182 spots in bracketing standards. Pits placed in domains containing organic matter yield a significantly higher count rate, and data from these pits were excluded from the data set (5 of 206 δ^13^C measurements in otoliths discarded). A complete table of data is included as online Table S2 in File S1. 

 Finally, 37 otoliths were analyzed with laser ablation ICPMS (LA-ICPMS). For this, a New Wave UP193 laser ablation unit was coupled to the ICPMS to ablate solid material from polished otolith sections. The parameters were set to 70% power, 35 μm spot size, 3 μm/s travel time, 10 Hz. The in-house otolith standard as well as a standard developed by the U.S. Geological Survey, MACS-3 [35], were used both for calibration and to correct for instrument drift. For most runs, transects were set to traverse a pre-defined growth axis (usually ventral or anterior) from the core to the outer edge. In some cases, two transects on different axes were made. Precision on standards was 8-10% for Ca and usually higher for other elements; hence, the data were smoothed with a 5-point interval. However, when comparisons were made between SIMS and other methods (both SXFM and LA-ICPMS), care was taken to localize the data as closely as possible to each other on the same growth bands (i.e., without smoothing). Seven minor or trace elements (Ba, Cu, Mg, Mn, Na, SrSr, and Zn) as well as Ca could be quantified.

 Migratory status was determined by whether a marked change was observed in otolith chemistry (Figure 3, see above). The putative first emigration from the Little Colorado River to the mainstem was identified both by the chemical signature on the otolith as well as a marked narrowing of daily growth increments [23]. This latter was due to movement into colder water, which slows both somatic and otolith growth. The temperature of the mainstem was consistently lower than the Little Colorado River (mean ΔT = 6.9 °C; Table S3 in File S1). Temperature, rather than food availability, most strongly affects the circadian pattern of daily increment deposition [36], so much so that hatcheries routinely batch mark otoliths by manipulating temperatures slightly upward to induce a broad growth band or downward to induce a narrow band [37]. Thus, the combination of shifts in otolith chemistry and narrowing of daily growth increments was interpreted as emigration from the Little Colorado River to the mainstem. In addition, where chemical analysis was lacking but otoliths were readable, we determined the residency status of an additional 29 individuals by widths of daily increments alone. 

## Results

### Water chemistry

**Figure 4:**
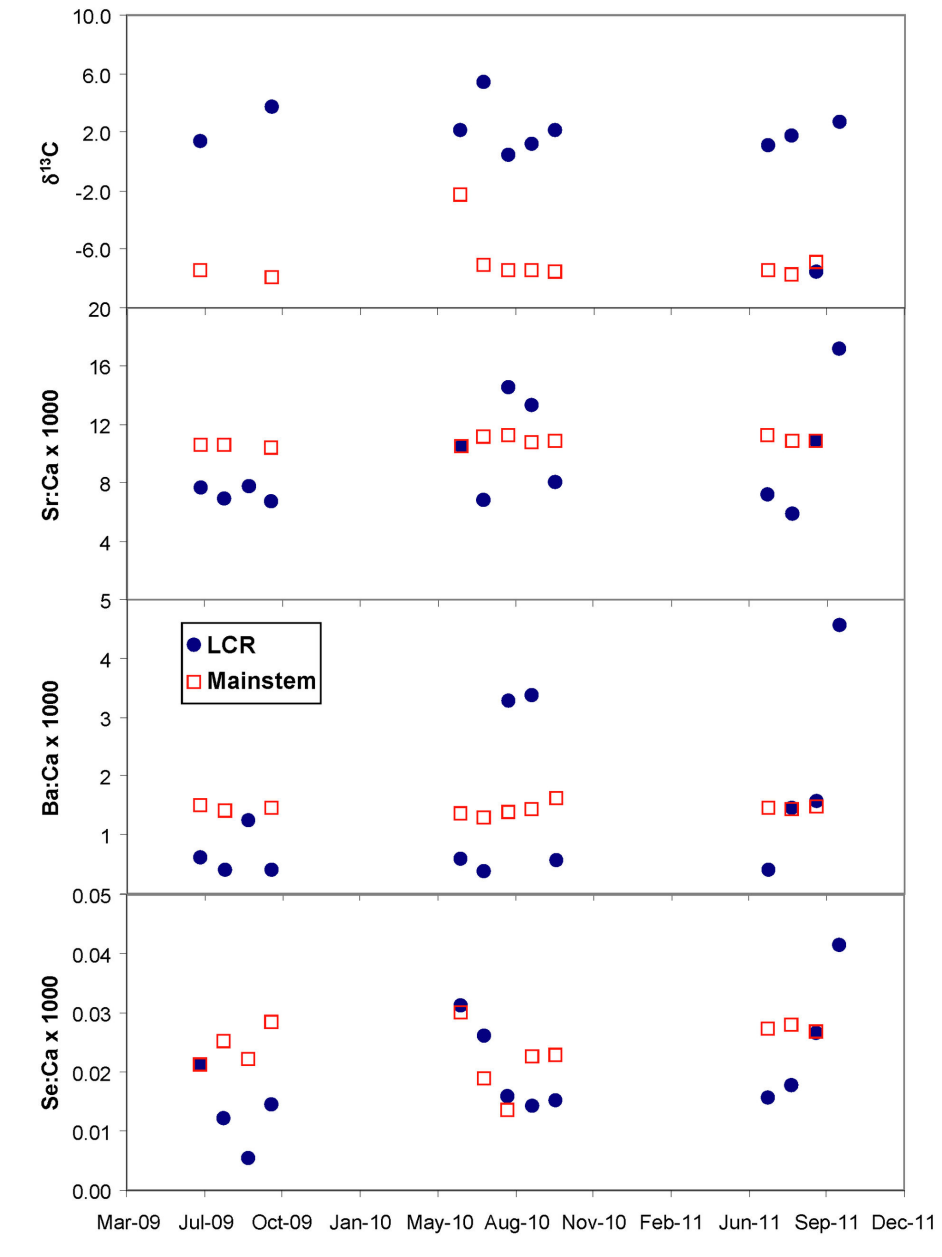
Comparison of water chemistry in the mainstem just above Little Colorado River, and Little Colorado River at Boulders, 2009 - 2011. Each year has a single value for July - October.

Water sampling over the three years of study revealed differences between the geochemistry of the Little Colorado River vs. the mainstem (Table 2, Figure 4). Barium, selenium, and strontium in ratio to calcium were lower (but not statistically different (p > 0.05)), and δ^13^C was higher (p < 10^-6^), in the Little Colorado River. Importantly, the variability of the Little Colorado River chemical parameters was far greater than was the case for the mainstem Colorado. Examination of the data (Figure 4) shows that Little Colorado River Ba:Ca, Se:Ca, and SrSr:Ca were lower on 8, 11, and 9 of 13 sampling events, respectively, and δ^13^C was enriched on all but one of 11 sampling events (δ^13^C analysis was not done in July - September 2009). Most of the carbon was in the form of DIC, but this fraction was larger (0.97 ± 0.01 S.E., N = 10) in the Little Colorado River than in the mainstem (0.89 ± 0.01 S.E., N = 9). The difference between δ^13^C at the two sites averaged 9.3 ± 1.2 ‰ (S.E.)(Table S1 in File S1), with Little Colorado being higher.

**Table 2:** Means (± s.e.) of barium, selenium, and strontium relative to Ca, and δ^13^C collected over 3 years (May 2009 – October 2011).

River	Ba:Ca x 10^-3^	Se:Ca x 10^-3^	Sr:Ca x 10^-3^	δ^13^C, ‰
Little Colorado River	1.38 (0.36)	0.019 (0.002)	9.34 (0.92)	0.59 (1.24)
Colorado mainstem	1.43 (0.03)	0.024 (0.002)	10.80 (0.08)	-8.87 (0.49)

Notes: Little Colorado River includes a special collection made in June 2010, which investigated longitudinal variation in water chemistry of that river. δ^13^C is a weighted average of DIC and DOC. Little Colorado River trace elements N = 14 events; Colorado mainstem elements N = 13 events; Little Colorado River δ ^13^C N = 10; Colorado mainstem δ^13^C N = 9.

Because the monsoonal season for the region occurs in summer to early fall and generates flashy precipitation events in the Little Colorado River well above its base flow (Figure S2 in File S1), high flow events can rapidly and radically alter chemical conditions there. The discharge of the mainstem, on the other hand, is far more constant as it is controlled release water from Lake Powell and is 2-4 orders of magnitude greater than that of the Little Colorado River. The trace SrSr:Ca and Ba:Ca ratios in the Little Colorado River were inversely proportional to the fraction of a given month with flows less than threshold values of 6.5, 8, and 10 m^3^/s (used as proxies for base flow and near base flow), and δ^13^C was positively, though less strongly, correlated with these thresholds (Figure S3 in File S1). Little relationship was shown between Se:Ca ratios and thresholded flow frequencies (Figure S3 in File S1). These results suggest that high flow events tend to release more strontium and barium from the watershed, and that the same events tend to dilute the travertine δ^13^C with meteoric water. The high variability in the relationships also suggests that the concentrations may depend on which part of the watershed becomes wetted in specific flow events. However, we note that Ba:Ca and SrSr:Ca were lowest, and δ^13^C highest, when flows were < 10 m^3^/s. Given that over the study period, the Little Colorado River daily discharge was less than 10 m^3^/s 84% of the time, we conclude that differences are large enough between the two rivers to discern distinct differences that would appear in otolith chemistry. We also note that water sampling was conducted during monsoonal months and thus disproportionately sampled during or shortly after high flow events; on the other hand, the humpback chub live continuously in the environment, incorporating ambient chemistry of whichever river they are in at the time. 

### Otolith chemistry

Carbon stable isotopic ratios in the otoliths showed patterns consistent with residency in both the Little Colorado River and the mainstem Colorado River. Small (< 30 mm) fish collected at Boulders Camp in the Little Colorado River, 3 km upstream of its confluence with the mainstem, had mean δ^13^C values of -2.5‰ ± 0.6 (95% confidence interval) and did not differ significantly from values in migrant fish that were classified as residency in the Little Colorado River (-3.2‰ ± 0.6). The values for portions of migrant fish otoliths identified as residency in the mainstem were considerably depleted in ^13^C (δ^13^C = -11.3‰ ± 0.6). The difference between the otolith mainstem signature and the mean of the two Little Colorado River signatures (-2.9‰) is 8.3‰, remarkably close to the difference observed in water from the two sources.

**Figure 5:**
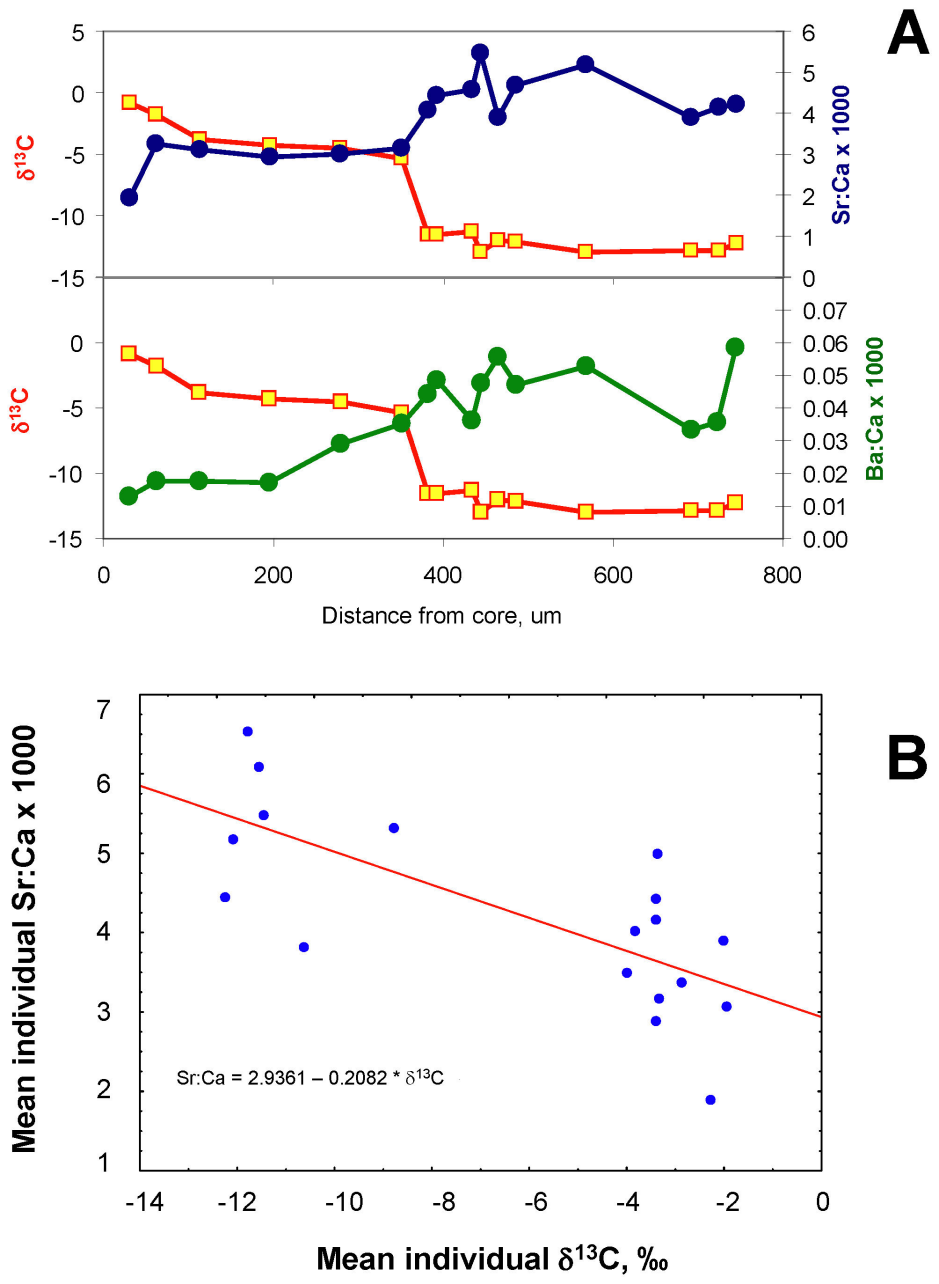
A. **Concordance of trace elements SrSr:Ca and Ba:Ca to δ^13^C in an otolith**. Carbon isotopic ratio shifts abruptly with habitat shift, whereas trace elements shift more gradually. B. Regression of SrSr:Ca on δ^13^C.

 The change in δ^13^C(otolith) from Little Colorado River to mainstem signature in otoliths tended to be abrupt, and could occur on the order of a day or two (Figure S4 in File S1). In contrast, trace elemental shifts tended to be more gradual (Figure 5). A linear regression of SrSr:Ca on δ^13^C was significant (R^2^ = 0.53, p < 10^-4^). Ba:Ca (outside of the core, see below) was also inversely related to δ^13^C but the relationship was more scattered (R^2^ = 0.15, p < 0.1).

**Figure 6:**
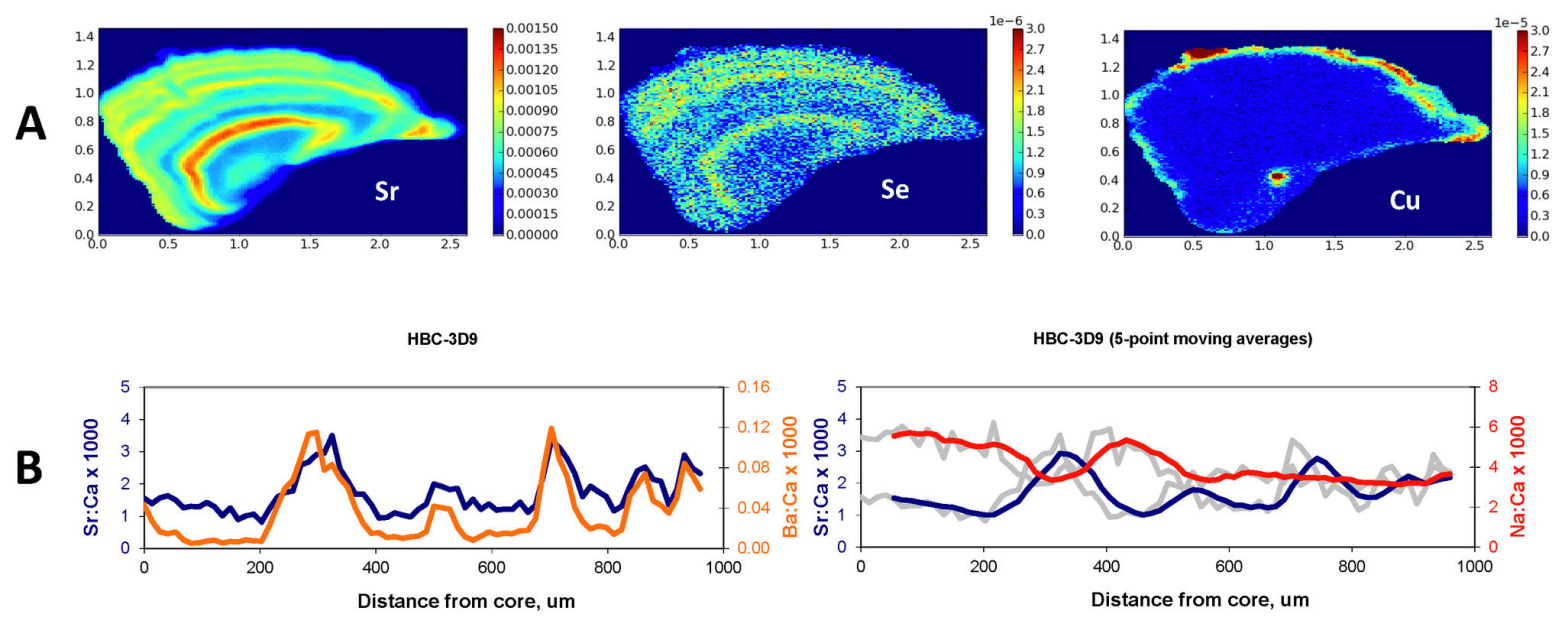
A. **Trace elemental maps generated by SXFM**. Note the concordance of SrSr and Se and the elevated Cu in the core region. B. Trace elemental transects of SrSr, Ba, and Na in ratio to Ca measured with LA-ICPMS. Note qualitative similarities of SrSr:Ca and Ba:Ca to the patterns in the trace elemental maps, and the inverse relationship of Na:Ca to SrSr:Ca up to approximately 550 microns.

 Despite the weak relationship of trace elements to δ^13^C, in some individual fish there were strong concordances among trace elements. For example, specimen HBC-3D9 had strong concordance in SrSr, Se, and Ba (Figure 6). We interpret the peaks in these elements as residency in the mainstem, and lower values as residency in the Little Colorado River. In contrast, sodium, which is high in the Little Colorado River [38], is elevated during this fish’s first growing season (summer), drops in the first winter, rises in the second summer, drops in the second winter, and then stabilizes (Figure 6B, right panel). We interpret this as further evidence of the fish spending the first two summers in the Little Colorado River and the first two winters in the mainstem. We hypothesize that the lack of dynamic pattern in the remainder of the Na:Ca transect may be due to physiological stabilization of ionic regulation, despite further movements between the two rivers. 

**Figure 7:**
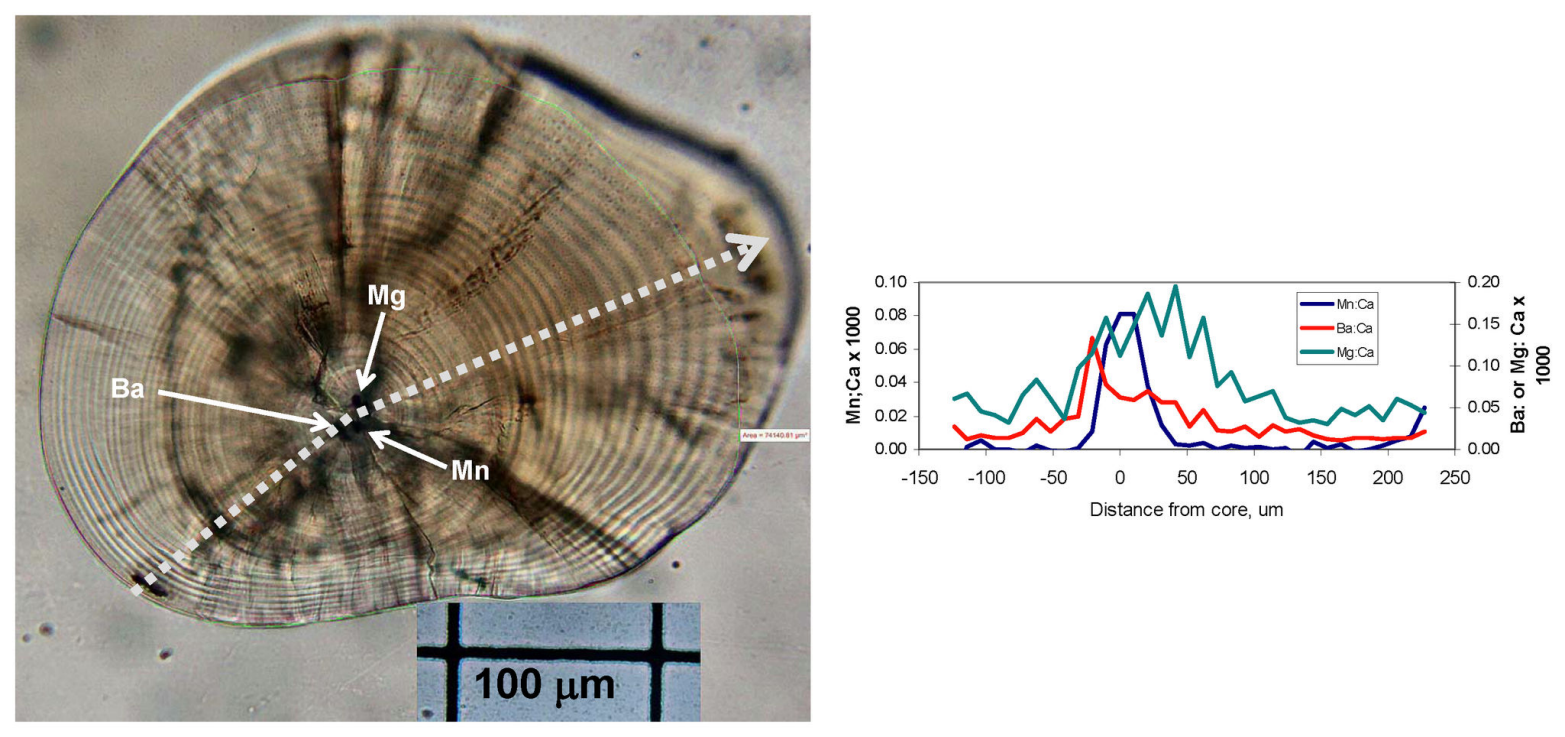
Otolith from a 25-mm, 30-day-old humpback chub captured in the Little Colorado River, June 2010. Note the presence of three primordia (arrows) that appear to be elevated in Ba, Mn, and Mg respectively. Dotted line shows direction of laser track but its width is not to scale.

 A number of otoliths had elevated concentrations of several trace elements in their core regions (Figure 7). These appear to be located in primordia or nucleation points within the core; a number of otoliths had visible multiple primordia. Most were detected with LA-ICPMS as SXFM is not sensitive to some of the elements due to interferences or low mass number. Up to five different trace elements (Ba, Cu, Mg, Mn, and Zn) were simultaneously detected as elevated in cores, often with a concomitant drop in SrSr concentration. Of 32 otoliths for which core analysis was of sufficient quality (i.e., the core was intact and exposed at the specimen surface), 7 contained all five elements (“Group 1”), 17 showed peaks in Ba, Mg, and Mn (“Group 2”), 2 had peaks in Ba, Mg, Mn, and either Cu or Zn (“Group 3”), and 6 had other combinations including only Mg, Mg and Mn, Ba and Cu, and a single individual had a sodium spike and no other elevated metals (“Group 4”). Analysis of variance revealed no effect on groups of capture location or year of birth; however, very few were captured away from the vicinity of the Little Colorado River/mainstem region. 

### Otolith-derived estimates of demographic events

**Figure 8:**
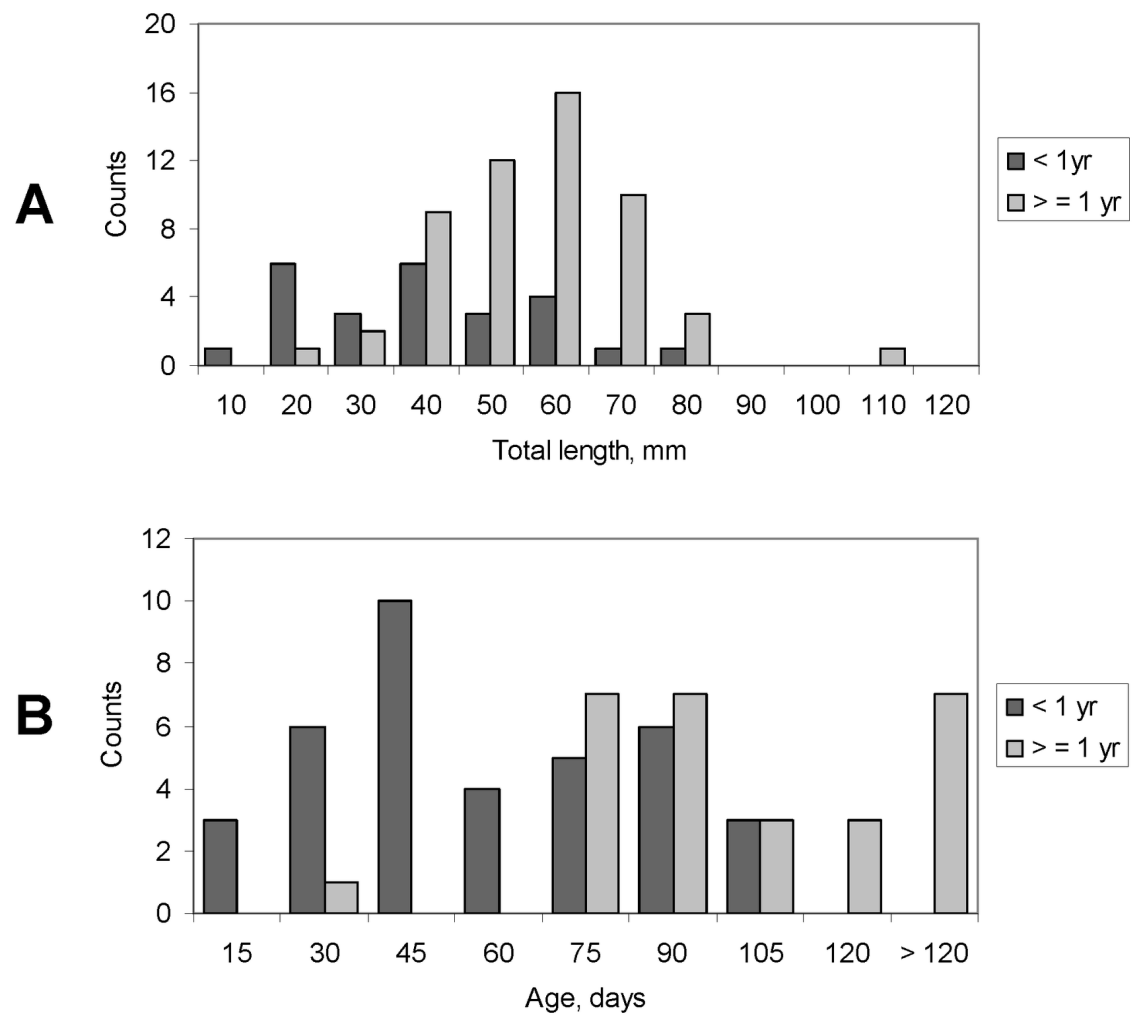
Frequency histograms of back-calculated humpback chub size (A) and age (B) at egress from the Little Colorado River, separated by age at time of capture.

77 otoliths were assessed for size at egress into the mainstem Colorado River. Back-calculated size at emigration ranged from 9.5 to 107.4 mm TL with a mean of 46.1 mm ± 2.1 S.E. The smallest-sized migrant had 19 broad daily increments, followed by a notable check and smaller daily increments, but eventually broadening out again even while the δ^13^C values remained depleted (Colorado River signature; Figure S4 in File S1). Size at egress differed significantly (p < 0.001) depending on whether the fish was a young-of-the-year or older. Mean size at egress of fish < 1 year at capture was 35.5 mm ± 3.6 s.e. (N = 25) whereas fish ≥ 1 year averaged 51.0 mm ± 2.2 s.e. (N = 52; Figure 8A). Generally, most individuals’ growth rates decelerated markedly upon entry into the mainstem, reflected in very tight otolith growth bands (see 23). 

Age at egress determinations were made on 65 fish. Age at first egress averaged 79.7 ± 6.1 (S.E.) days, though one was estimated to have been approximately 250 days old and emigrated at the end of its first winter. As with length at egress, age at egress differed significantly between fish less than or ≥ 1 year at capture (p < 0.001; Figure 8B). Young-of-the-year that emigrated were on average 51.2 ± 4.4 days at egress (N = 37); fish 1 year and older were 100 ± 7.8 days old at egress (N = 28). Age and length at first egress were strongly correlated and were fit with a linear regression (Age at egress = -7.95 + 1.96 (TL at egress), R^2^ = 0.61, p < 10^-4^ (Figure S5 in File S1). 

**Figure 9:**
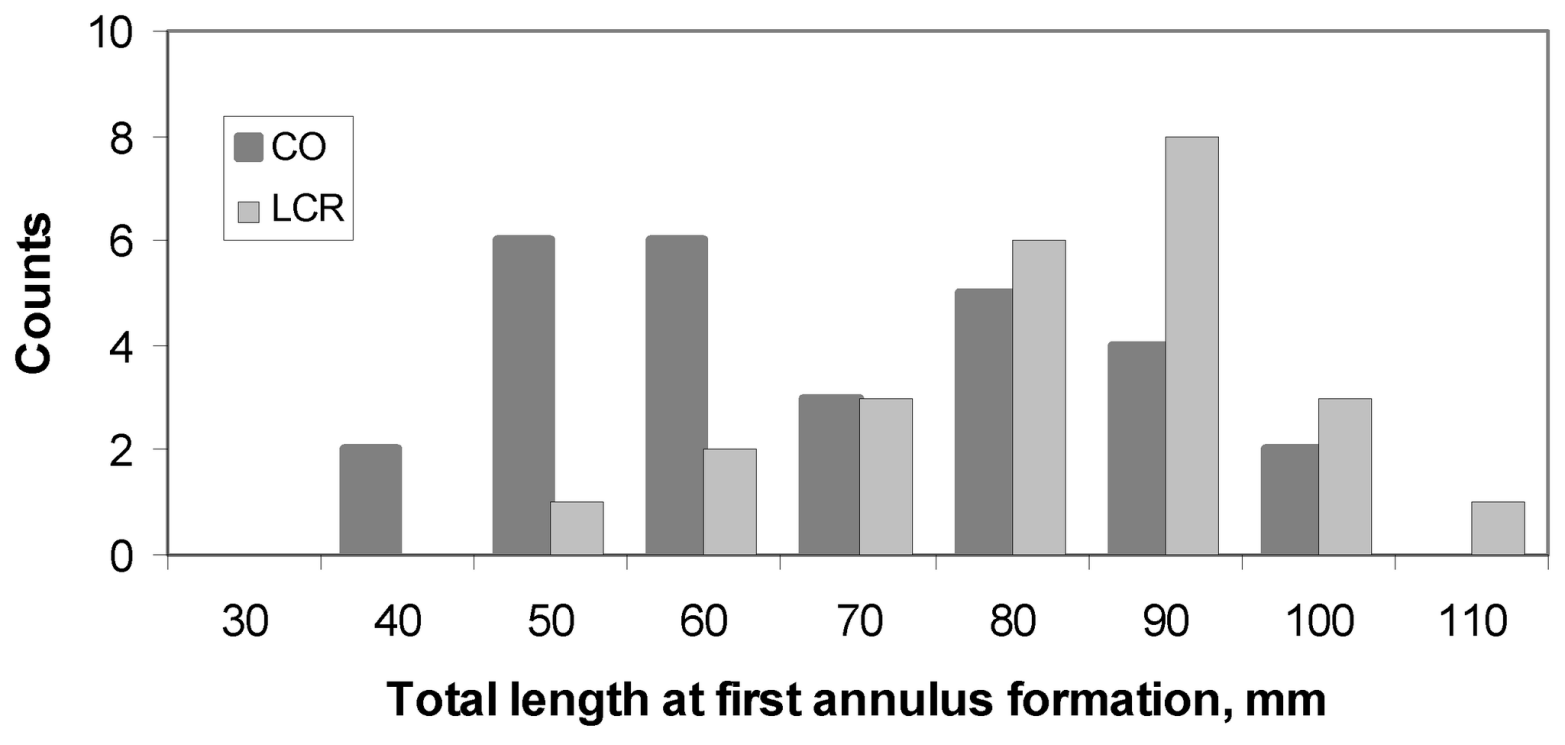
Back calculated lengths at first annulus formation for humpback chub captured in the Little Colorado (Little Colorado River) vs. **Colorado (CO) rivers**.

 Total length at first annulus formation (Age 1) varied between the two rivers (Figure 9). Specifically, length at Age 1 was unimodal in the Little Colorado River-caught fish, bimodal in the mainstem-caught fish, and bimodal overall. Mean length at Age 1 for fish captured in the Little Colorado River was 78.2 + 3.3 mm. Modes for the Colorado-caught vs. overall bimodal distributions were close (Table 3). 

**Table 3:** Results of bimodal analysis of back-calculated total lengths at Age 1.

**A. All fish:**					
Mode	p_i_	Mu	sigma	Chi^2^	PrPr(Chi^2^)
1	0.324 (0.11)	50.4 (3.51)	6.71 (2.68)	0.215	0.99
2	0.676 (0.11)	80.4 (2.97)	10.24 (2.21)		
**B. Colorado mainstem-caught fish:**					
Mode	p_i_	Mu	sigma	Chi^2^	PrPr(Chi^2^)
1	0.507 (0.15)	49.9 (3.59)	6.874	0.59	0.90
2	0.492 (0.15)	79.04 (4.94)	9.496		

p_i_ = proportion of data falling under the mode; Mu = modal mean; sigma = spread.

Standard errors in parentheses.

**Figure 10:**
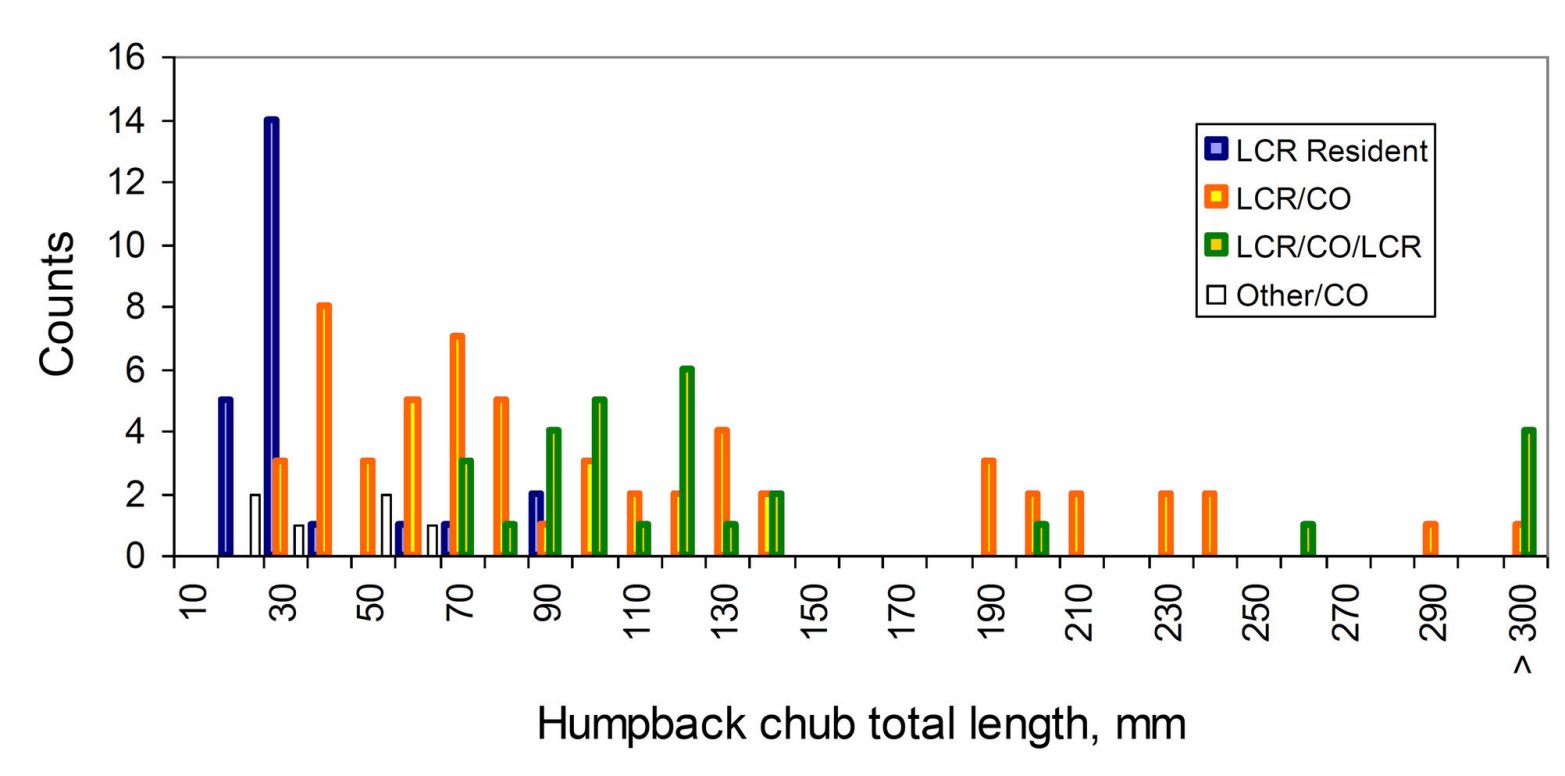
Number of humpback chub (y-axis) by size (x-axis) demonstrating specific residency patterns as determined from otolith microchemistry or growth analyses. “LCR Resident” are fish that were born in the Little Colorado River and were captured there. “LCR/CO” are fish that were born in the Little Colorado River and then emigrated to the Colorado River mainstem and were captured there. “LCR/CO/LCR” are fish that emigrated into the mainstem but were re-captured in the Little Colorado River. Finally, “Other/CO” are fish that were born in an unknown location, but then determined to be living in the Colorado River mainstem.

 We were able to assess provenance and recent residency of 126 humpback chub from 15-408 mm TL (Figure 10). We found that 95% of the fish we assessed were born in the Little Colorado River and the remainder from an unidentified location. Of these 126 fish, 23% (N = 30) were born in the Little Colorado River, then used the mainstem Colorado River for some portion of their life, and subsequently returned to the Little Colorado River where they were captured. An additional 50% (N = 66) fish were born in the Little Colorado River and remained in the mainstem after egress while 18% (N = 24) were Little Colorado residents. The remaining 5% (N = 6) were not clearly identifiable with respect to provenance. Based on examination of growth patterns it appears that these fish were most likely born in the mainstem in a location that is atypical in terms of chemistry and temperature to the surrounding water such as a warm spring location [23]. 

## Discussion

Water chemistry in the mainstem Colorado River was characterized by constancy, whereas our sampling of water chemistry in the Little Colorado River showed considerable variability as it transitioned between its “blue water” baseflow to its turbid, high flow states. This variability was driven by high flow events that were disproportionately sampled relative to their actual frequency of occurrence in the Little Colorado River. When accounting for flow frequency, water chemistry differed sufficiently between the Little Colorado and mainstem Colorado rivers to provide reliable end member markers that could be taken up and quantified in humpback chub otoliths. 

 Of the markers investigated, δ^13^C discriminated most strongly between rivers and was faithfully reflected in otoliths. Carbon stable isotope ratios become elevated in the Little Colorado River due to degassing of deep groundwater as it bubbles out of springs in the system, precipitating travertine at the same time. As is typically the case in lotic travertine systems [22], δ^13^C increased downstream from Blue Spring (Limburg et al., in preparation). Carbon isotopic uptake in humpback chub otoliths was rapid; we detected changes of up to ~ 9‰ on the order of 1-2 days in closely-spaced spot measurements which suggests that carbon signatures should exist in otoliths to delineate spatial location of a fish within a few days of the fish moving from the Little Colorado River to mainstem or vice-versa. In an experimental study, ^14^C uptake from labeled ambient DIC was measurable in goldfish *Carassius auratus* otoliths as soon as one hour following incubation [39]. These investigations also found that 75% of the carbon incorporated in otoliths came from DIC with the remainder being metabolic; this was similar to the findings of 80% DIC derived otolith carbon in rainbow trout *Oncorhynchus mykiss* [40]. Metabolic C contributed 35 - 45% of otolith C in bluegill sunfish *Lepomis macrochirus* that were exposed to a whole-lake addition of ^13^C and the change in lake chemistry was transmitted to the otoliths in a few days [41]. Similarly, we assume that most of the C taken up by humpback chub otoliths derives from the DIC rather than from their food; however the food sources should also be isotopically labeled by the DIC [42]. When examining data from YOY that were assayed, the δ^13^C appeared to be approximately 4 to 5 per mille depleted compared to source water analyzed from the same general time period. Solomon et al. [40] reported a small, net depletion of otolith δ^13^C relative to sources, and found this was due to a large depletion when C entered the bloodstream, followed by enrichment when C was incorporated into the otolith. 

Although δ^13^C is a superior tracer of fish provenance and movement in this system, in practice it is a difficult and costly analysis, both in water and otoliths. We therefore sought [23; this study] complementary tracers that are easier and more cost-effective to use. In this regard, strontium:calcium (Sr:Ca) ratios appear to be the most reliable choice. This as well as other trace elemental markers (Ba:Ca, Se:Ca) in chub otoliths appear to lag by perhaps 1-2 weeks relative to carbon stable isotopes, and the transition is more gradual as well. This may in part be due to an averaging effect of the larger beam sizes used in the trace elemental analyses. Thus, we over-estimate slightly our age and size at emigration when using trace elemental markers alone; however, this overestimation should be evenly distributed amongst emigrants. Others have also noted the gradual incorporation of SrSr:Ca into otoliths [e.g., 43]. 

Sodium is another potential tracer of juvenile humpback chub residency in the Little Colorado River. This is referred to as a “salt river,” and Hopi Indian traditions include a pilgrimage along the Salt Trail [44]. Although present in moderately high concentrations in otoliths, sodium has generally been assumed to be under such great physiological control that it would serve little value as a provenance tracer [45]. However, the very high concentrations of sodium in the Little Colorado River may cause an increase of otolith Na:Ca during the juvenile period, when growth is rapid. Hoff and Fuiman [46] found a significant decline in Na:Ca ratios in juvenile red drum *Scianops ocellatus* exposed to increasing salinities. The relationship of high dissolved sodium to otolith Na:Ca doubtless is constrained by physiology and phylogeny (drum are scianids and chubs are minnows), but bears further investigation in humpback chub.

In a similar vein, we found that a number of different trace elements were highly concentrated in the primordia of chub otoliths. Manganese has often been cited as under maternal control as its presence in otolith cores has been found across different taxa [e.g., 47,48] but not in all species [e.g., 32]. In the present study, we found that 7 different elements could “spike” in the otolith core, and up to 5 of them more or less simultaneously. However, careful examination of lasered specimens showed that spikes of different elements could occur in adjacent primordia (Figure 7). It is likely that primordia chemistry is determined by maternal deposition of trace elements in eggs. Future work should further explore the causes and whether there are patterns of geographic variation within the Colorado River system; for example, eggs could be collected from females in different parts of the Grand Canyon and analyzed for trace elements.

How does an understanding of these chemical tracers and otolith growth of humpback chub help in our understanding of the population ecology of this species? We suggest two key findings: (1) age and size at egress of juvenile humpback chub from the Little Colorado River into the mainstem differed between young-of-the-year and older individuals, and (2) size at age-1 of humpback chub differed between fish captured in the Little Colorado River vs. the mainstem. Little Colorado River humpback chub size at age-1 was distributed about a unimodal peak, whereas mainstem fish had a bimodal peak (Figure 8). The larger of the bimodal peaks from the Colorado River matched closely the mode of the distribution of fish from the Little Colorado River. This suggests that this larger of the two modes observed in the mainstem may represent fish that remained in the Little Colorado River for a longer period of time before emigrating to the Colorado River. 

We found that most of the young-of-the-year humpback chub (27 of 37) we assessed were estimated to emigrate from the Little Colorado River during June-August^1^, when Little Colorado River flows can be at some of their lowest levels of the year depending on when monsoon rains begin. As an example, of these 37 fish that were captured in the mainstem that we were able to estimate month of egress, 20 of these came from 2009. When daily flows in the Little Colorado River are examined for 2009 (USGS gage 09402300), values from May- December are generally similar between 6-8 m^3^/s, with three small monsoonal storm peaks (May 24 at 23.9, July 21 at 11.2, and September 14 at 15 m^3^/s). Of the 20 fish in our sample that emigrated from the Little Colorado River to the mainstem, 8 emigrated in August, 4 each in June and July, 3 in May, and 1 in April – all on dates having low flow conditions. Granted, our sampling was uneven and constrained by permit requirements, so we cannot state that this is a dominant behavior. Nevertheless, it demonstrates that emigration is not necessarily dependent on flood pulses, and that juvenile humpback chub may emigrate based on some other cue, potentially humpback chub density in the Little Colorado River or some other ecological or environmental phenomenon.

Our findings suggest a size advantage at their first birthday for fish that remain in the Little Colorado River for longer time periods, and that this size advantage may persist when these fish egress into the mainstem Colorado River. For humpback chub, survival is thought to be strongly influenced by the fish age and size and this relationship is assumed in the key stock assessment framework developed for this species [9]. Larger body size is known to offer many survival advantages including improved winter survival related to higher lipid accumulation [49], greater range of prey resources available [50], and improved swimming ability and predator avoidance [51] ultimately potentially leading to improved fitness for these surviving individuals [52 - 54]. All of these advantages may be important for juvenile humpback chub to survive in the mainstem Colorado River under current conditions. In the post-Glen Canyon Dam environment, invertebrate fish prey resources may be altered such that fish now must broaden their diets [55] even while prey availability declined. Additionally, cold water temperatures may impair swimming ability for smaller fish [10,17], leading to increased predation risk from non-native predators [11]. These survival constraints could be mitigated by larger size at egress from the Little Colorado to Colorado rivers.

Growth patterns of juvenile humpback chub (these same fish) were documented from tag recaptures in the Little Colorado and mainstem Colorado River in response to experimental flow retreatments in the mainstem [56 and unpublished data]. Based on growth rates estimated from tag-recaptured fish, in order to maximize growth, juvenile humpback chub should spend spring and summer in the Little Colorado River and emigrate to the mainstem Colorado River during winter when growth declined. Evidence was found of juvenile humpback chub moving from the Little Colorado River, to the mainstem, and then returning to the Little Colorado River from otolith microchemistry [23] and efforts are underway to assess these and other movement patterns by juveniles using tags (W. Persons, U.S. Geological Survey, Grand Canyon Monitoring and Research Center, *pers. comm.*). This type of movement pattern would potentially maximize growth from a temperature perspective but it is unknown if prey availability follows the same spatial and temporal pattern between the Little Colorado and mainstem Colorado Rivers. In our current study, this behavior was rare amongst juveniles (

< 3%); it may be necessary to increase the sample size, including the spatial and temporal distribution of samples, to obtain a more reliable perspective on these types of fine-scale movement patterns between these systems. This study is based primarily on incidental mortalities which may introduce unknown bias into our interpretation. However, given the Endangered status of humpback chub, the risk to the population from directed sampling of larger numbers of humpback chub was unknown. This risk is now better quantified and can be considered for future directed sampling for humpback chub otoliths to address additional research and management questions [18].

While it is well documented from taPrPrgging studies that adult humpback chub migrate between the mainstem Colorado River and Little Colorado River for spawning [57], movement patterns of juveniles between the mainstem and Little Colorado rivers prior to spawning is not as well studied and has been difficult to assess due to low capture probabilities of tagged fish and limited sampling in the mainstem Colorado River for juvenile fish. Collectively our results, including Hayden et al. [23], suggest that juvenile humpback chub can and do utilize both the Little Colorado and mainstem Colorado rivers. Additionally, ongoing work suggests a mechanistic framework for why it may be thermally advantageous for juvenile fish, in terms of improved growth and survival [56], to use both rivers. This is important because management efforts have been directed at improving conditions in the mainstem Colorado River to promote survival of juvenile humpback chub. These efforts include experimental flows to improve survival [56], modify habitats [58,59], and removals of non-native species [14,11]. In contrast, limited conservation actions have been made in the Little Colorado River since critical habitat designation [60]. In view of the fact that special areas such as Blue Springs and other groundwater sources still merit designation 20 years afterwards [61,62], additional conservation and protection of the Little Colorado River may be warranted. 

We cannot definitively address the robustness of our geochemical atlas across seasons, but rather must make inferences about the applicability of our collected data to other times of the year. That said, the weight of the evidence within humpback chub otoliths, in terms of combined chemistry and growth patterns [36], supports our contention that the markers are valid for assigning fish emigrant status. Together with retrospective estimates of size and age at key events (first egress and first birthday), our results support the hypothesis that chub remaining in the Little Colorado longer than ca. 2 months have improved chances of recruitment, and that those remaining even longer in the Little Colorado River grow larger by Age 1 and are thus better conditioned for survival in the mainstem Colorado. Depending on whether additional, systematic sampling of humpback chub occurs, other questions could be investigated, such as whether winter residency in one or the other river enhances or retards growth, how many winters are spent in one vs. the other river, what proportion of chub located in the Little Colorado River are true residents of that area, etc.

The largest question – how important *is* the Little Colorado River to humpback chub? – is answered easily as “very important.” There is limited evidence for successful spawning in other locations in this river reach other than the Little Colorado River [23,63] and the risk to this population from some sort of catastrophic event in the Little Colorado River has motivated management agencies to develop a series of actions to conserve this species including translocation efforts to other tributaries [64]. Additionally the key management guidelines for this species [65] specify coordinated efforts by conservation interests to expand research assessing the role of tributaries in contributing to the resilience of humpback chub populations. Our results help to provide a tool to meet this goal by creating a framework for assessing the contribution of recruits from the mainstem Colorado River and from tributary systems with different water chemistry signals. This information could be extremely useful in prioritizing conservation efforts for developing secondary spawning populations of humpback chub as specified in recovery documents [65].

## Conclusions

Our results confirming the use of the Colorado River as juvenile rearing habitat, coupled with the persistence of these juvenile cohorts for multiple years [56] suggests that, at least in recent years, the mainstem Colorado River has been suitable for juvenile humpback chub. This is a significant finding. It is likely that this rearing capacity, in addition that of the Little Colorado River [61] is a potential reason for the observed increases in juvenile recruitment and ultimately adult humpback chub documented in the Little Colorado River aggregation in recent years [66,61]. Overall this work clarifies the important role the mainstem Colorado River in Grand Canyon likely plays in the recovery of humpback chub populations. 

## Supporting Information

File S1 **Contains Figure S1–Figure S5 and Table S1–Table S3.** **Figure S1:** Photo of travertine formations in Little Colorado River. Credit: C. Finch. **Figure S2:** Discharge characteristics of the Little Colorado River near its confluence with the mainstem Colorado River, May 2009 – December 2012. A. Hydrograph. Lines represent the 6.5, 8, and 10 m3/s levels of discharge. Note the flashy storm hydrographs interspersed among very low base flow conditions. Data from U.S. Geological Survey National Water Information System (http://waterdata.usgs.gov/nwis). B. Flow frequency curve for Little Colorado River. C. Temporal patterns of flow frequencies less than 6.5 (blue), 8 (red), and 10 (green) m3/s in Little Colorado River. **Figure S3:** Linear regressions of (A) barium:calcium ratios, (B) strontium:calcium ratios, (C) selenium:calcium ratios, and (D) ™13C, all vs. the percent of discharges within a month that was less than specified threshold values of 6.5, 8, and 10 m3/s. **Figure S4:** Example of an otolith showing positions of ion microprobe ablations and corresponding δ13C data. The fish was collected on 23 July, 2010 in the mainstem; it was 24 mm TL and 63 days old. Note the rapid shift from elevated (Little Colorado River) to 13C-depleted values. **Figure S5:** Humpback chub total length (mm) vs. age (days) at egress from the Little Colorado River to the mainstem Colorado River. **Table S1:** Carbon isotopic ratios (δ13C, ‰) and C concentrations (ppm) in dissolved inorganic carbon (DIC), dissolved organic carbon (DOC), and concentration-weighted average δ13C at Boulder Camp, most downstream sampling site in the Little Colorado River, and the mainstem Colorado River upstream of the Little Colorado. Absolute difference in δ13C between the two sites is computed for co-occurring dates. **Table S2:** *In situ* carbon isotope analysis by SIMS (Secondary Ion Mass Spectrometry). **Table S3:** Mean (± 95% confidence intervals) monthly temperatures, 2008-2012, in the Little Colorado River (LCR) and the Colorado River mainstem (Mainstem), 2008-2012. Difference between means is given as ΔT.
(PDF)
