# Supplementary material for: Of Travertine and Time: Otolith Chemistry and Microstructure Detect Provenance and Demography of Endangered Humpback Chub in Grand Canyon, USA
Source: PLoS One. 2013 Dec 16;8(12):e84235. doi: 10.1371/journal.pone.0084235 (PMC3865257; doi:10.1371/journal.pone.0084235)
Supplement: File S1 — Contains Figure S1–Figure S5 and Table S1–Table S3. Figure S1: Photo of travertine formations in Little Colorado River. Credit: C. Finch. Figure S2: Discharge characteristics of the Little Colorado River near its confluence with the mainstem Colorado River, May 2009 – December 2012. A. Hydrograph. Lines represent the 6.5, 8, and 10 m3/s levels of discharge. Note the flashy storm hydrographs interspersed among very low base flow conditions. Data from U.S. Geological Survey National Water Information System (http://waterdata.usgs.gov/nwis). B. Flow frequency curve for Little Colorado River. C. Temporal patterns of flow frequencies less than 6.5 (blue), 8 (red), and 10 (green) m3/s in Little Colorado River. Figure S3: Linear regressions of (A) barium:calcium ratios, (B) strontium:calcium ratios, (C) selenium:calcium ratios, and (D) ™13C, all vs. the percent of discharges within a month that was less than specified threshold values of 6.5, 8, and 10 m3/s. Figure S4: Example of an otolith showing positions of ion microprobe ablations and corresponding δ13C data. The fish was collected on 23 July, 2010 in the mainstem; it was 24 mm TL and 63 days old. Note the rapid shift from elevated (Little Colorado River) to 13C-depleted values. Figure S5: Humpback chub total length (mm) vs. age (days) at egress from the Little Colorado River to the mainstem Colorado River. Table S1: Carbon isotopic ratios (δ13C, ‰) and C concentrations (ppm) in dissolved inorganic carbon (DIC), dissolved organic carbon (DOC), and concentration-weighted average δ13C at Boulder Camp, most downstream sampling site in the Little Colorado River, and the mainstem Colorado River upstream of the Little Colorado. Absolute difference in δ13C between the two sites is computed for co-occurring dates. Table S2: In situ carbon isotope analysis by SIMS (Secondary Ion Mass Spectrometry). Table S3: Mean (± 95% confidence intervals) monthly temperatures, 2008-2012, in the Little Colorado River (LCR) and the Colorado R [file pone.0084235.s001.pdf]

Supporting Information – Limburg et al.

Supporting Figures.

Figure S1. Photo of travertine formations in Little Colorado River. Credit: C. Finch.

Figure S2. Discharge characteristics of the Little Colorado River near its confluence with the mainstem Colorado River, May 2009 – December 2012. A. Hydrograph. Lines represent the 6.5, 8, and 10 m^3/s levels of discharge. Note the flashy storm hydrographs interspersed among very low base flow conditions. Data from U.S. Geological Survey National Water Information System (http://waterdata.usgs.gov/nwis). B. Flow frequency curve for Little Colorado River. C. Temporal patterns of flow frequencies less than 6.5 (blue), 8 (red), and 10 (green) m3/s in Little Colorado River.

Figure S3. Linear regressions of (A) barium:calcium ratios, (B) strontium:calcium ratios, (C) selenium:calcium ratios, and (D) $\delta^{13}C$, all vs. the percent of discharges within a month that was less than specified threshold values of 6.5, 8, and 10 m^3/s.

Figure S4. Example of an otolith showing positions of ion microprobe ablations and corresponding $\delta^{13}C$ data. The fish was collected on 23 July, 2010 in the mainstem; it was 24 mm TL and 63 days old. Note the rapid shift from elevated (Little Colorado River) to ^{13}C-depleted values.

Figure S5. Humpback chub total length (mm) vs. age (days) at egress from the Little Colorado River to the mainstem Colorado River.

Supporting Tables.

Table S1. Carbon isotopic ratios ($\delta^{13}C$, ‰) and C concentrations (ppm) in dissolved inorganic carbon (DIC), dissolved organic carbon (DOC), and concentration-weighted average $\delta^{13}C$ at Boulder Camp, most downstream sampling site in the Little Colorado River, and the mainstem Colorado River upstream of the Little Colorado. Absolute difference in $\delta^{13}C$ between the two sites is computed for co-occurring dates.

Table S2. *In situ* carbon isotope analysis by SIMS (Secondary Ion Mass Spectrometry).

Table S3. Mean (± 95% confidence intervals) monthly temperatures, 2008-2012, in the Little Colorado River (LCR) and the Colorado River mainstem (Mainstem), 2008-2012. Between-river differences are given as ΔT.

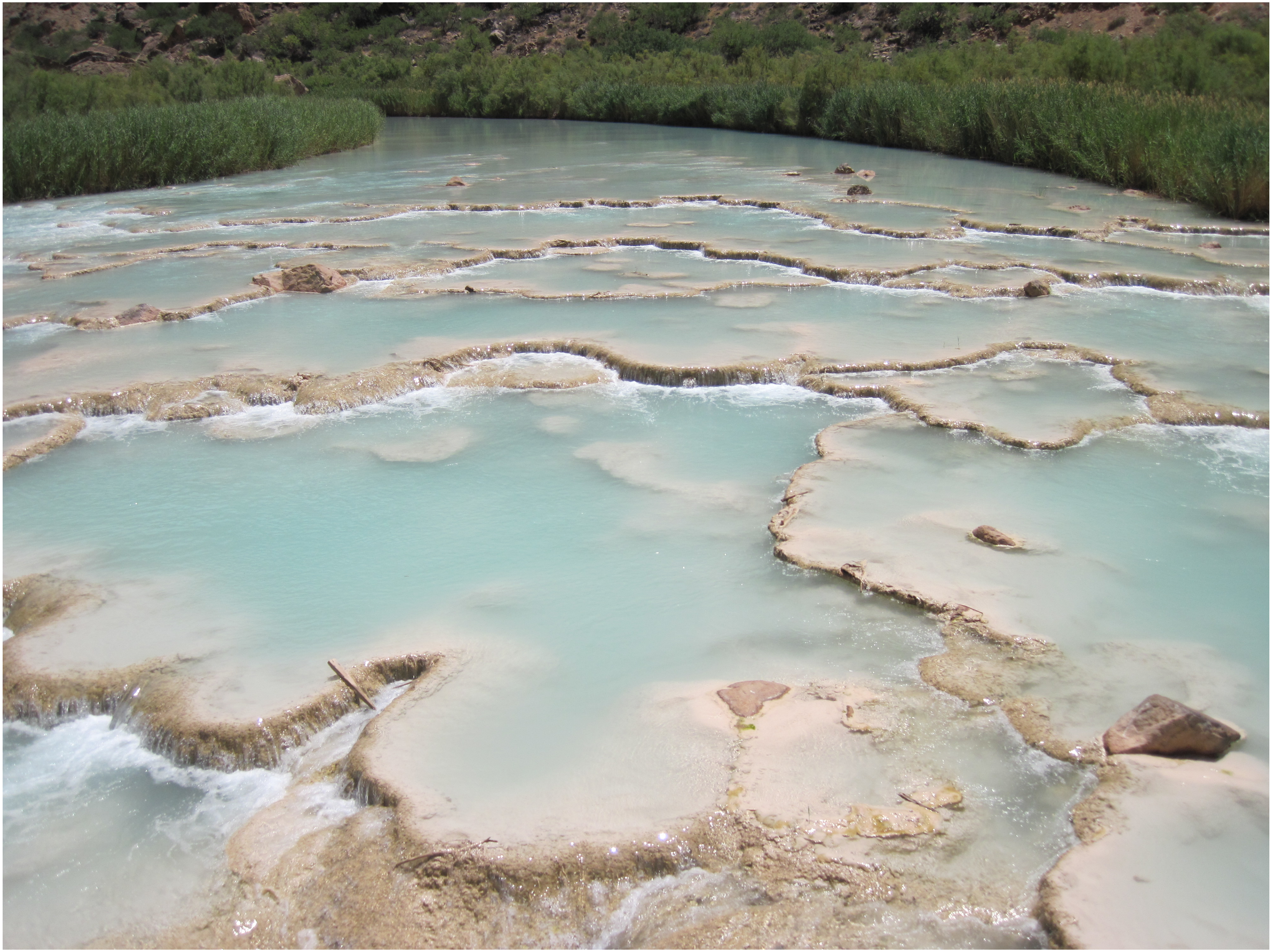

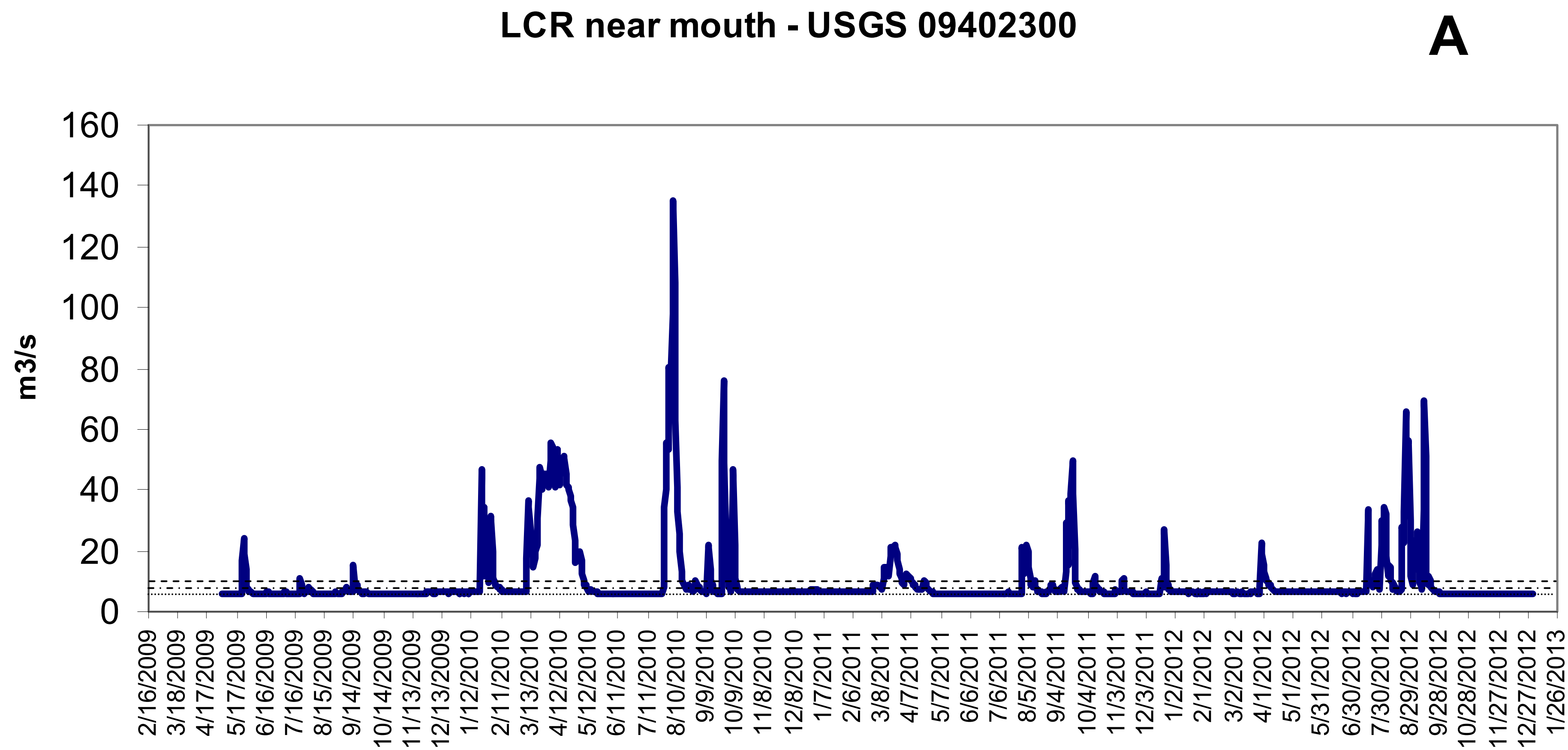
LCR near mouth - USGS 09402300
A
m3/s
160
140
120
100
80
60
40
20
0
2/16/2009
3/18/2009
4/17/2009
5/17/2009
6/16/2009
7/16/2009
8/15/2009
9/14/2009
10/14/2009
11/13/2009
12/13/2009
1/12/2010
2/11/2010
3/13/2010
4/12/2010
5/12/2010
6/11/2010
7/11/2010
8/10/2010
9/9/2010
10/9/2010
11/8/2010
12/8/2010
1/7/2011
2/6/2011
3/8/2011
4/7/2011
5/7/2011
6/6/2011
7/6/2011
8/5/2011
9/4/2011
10/4/2011
11/3/2011
12/3/2011
1/2/2012
2/1/2012
3/2/2012
4/1/2012
5/1/2012
5/31/2012
6/30/2012
7/30/2012
8/29/2012
9/28/2012
10/28/2012
11/27/2012
12/27/2012
1/26/2013

Figure S2

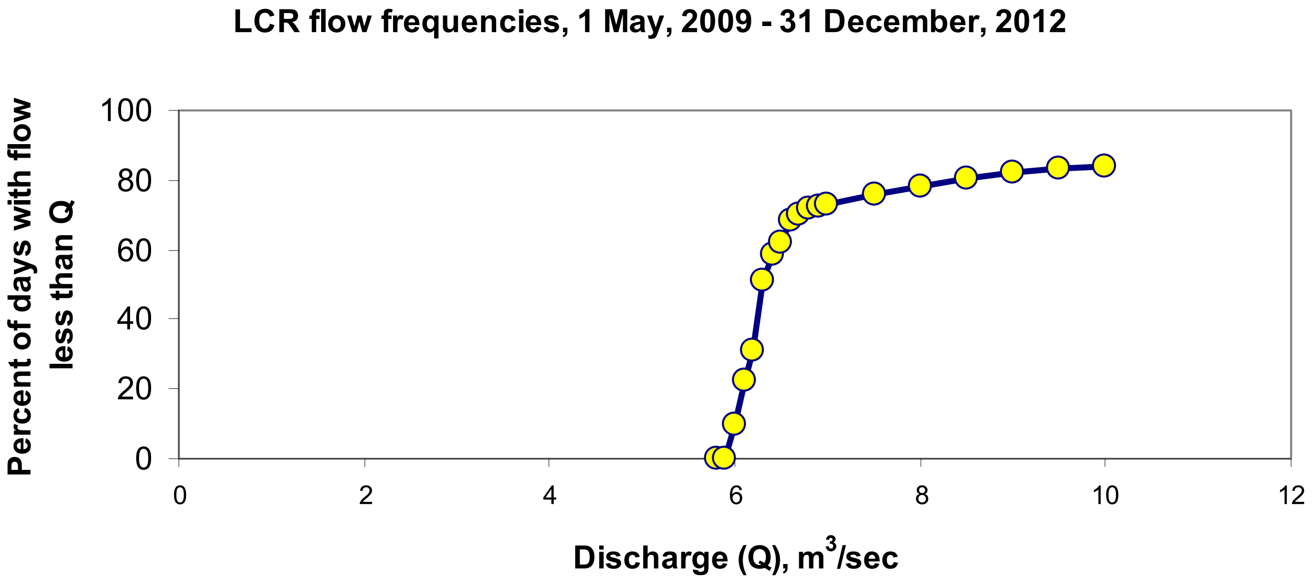

B

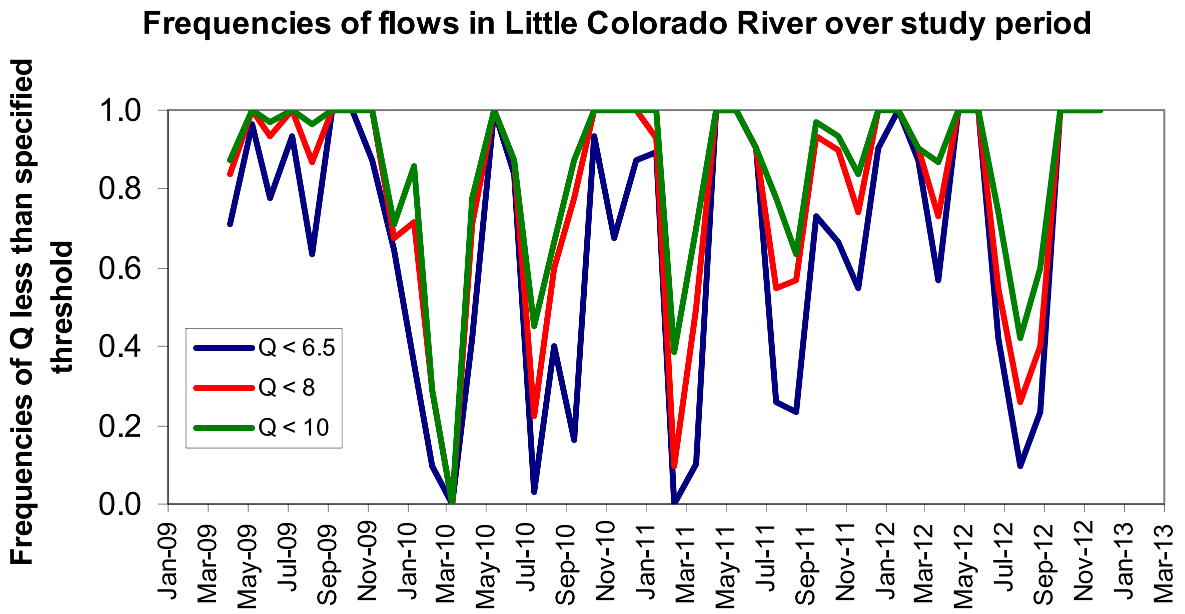

C

Figure S-2, continued

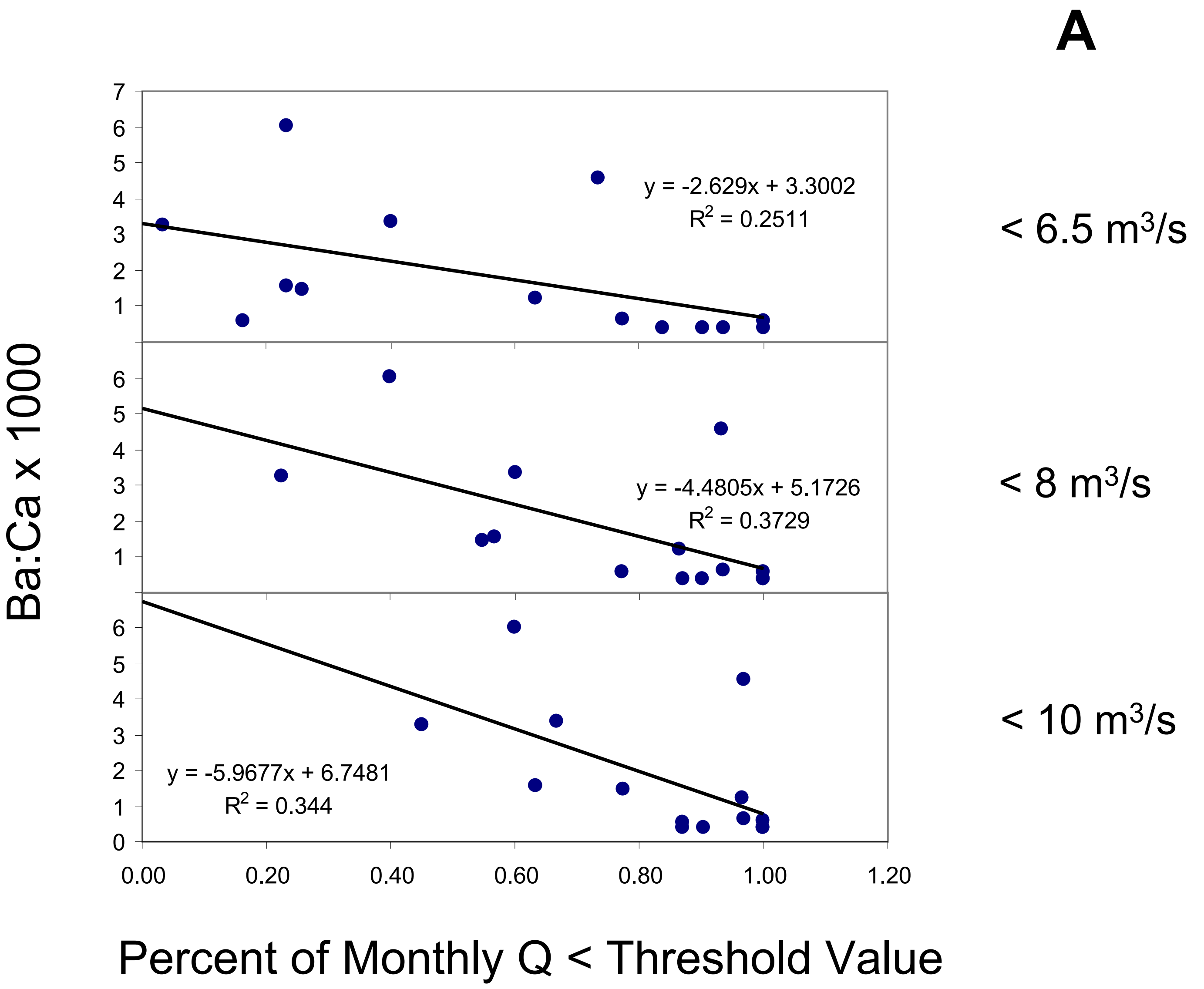

A
Ba:Ca x 1000
y = -2.629x + 3.3002
R2 = 0.2511
< 6.5 m3/s
y = -4.4805x + 5.1726
R2 = 0.3729
< 8 m3/s
y = -5.9677x + 6.7481
R2 = 0.344
< 10 m3/s
0.00
0.20
0.40
0.60
0.80
1.00
1.20
Percent of Monthly Q < Threshold Value

Figure S3

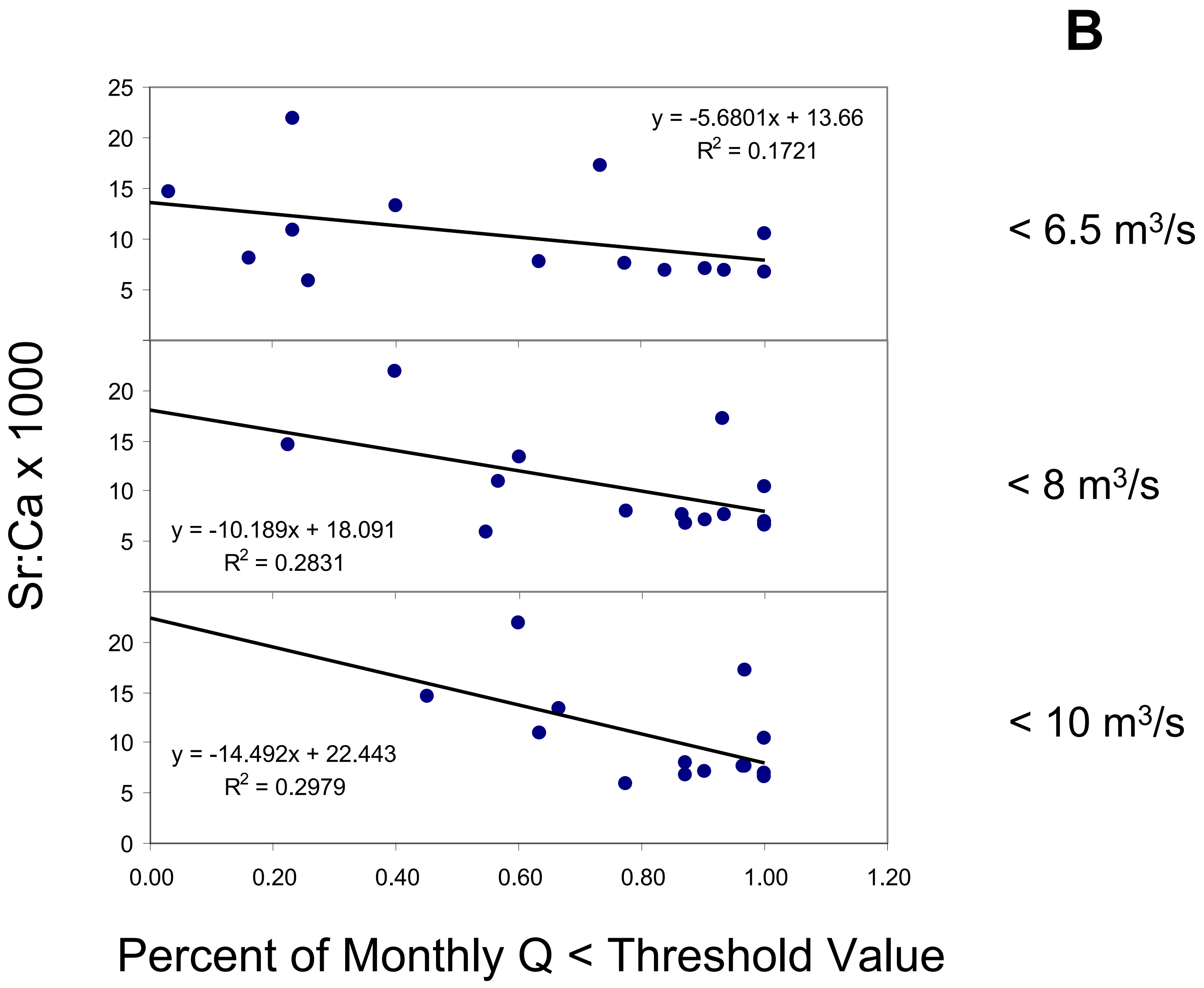

Figure S3, continued

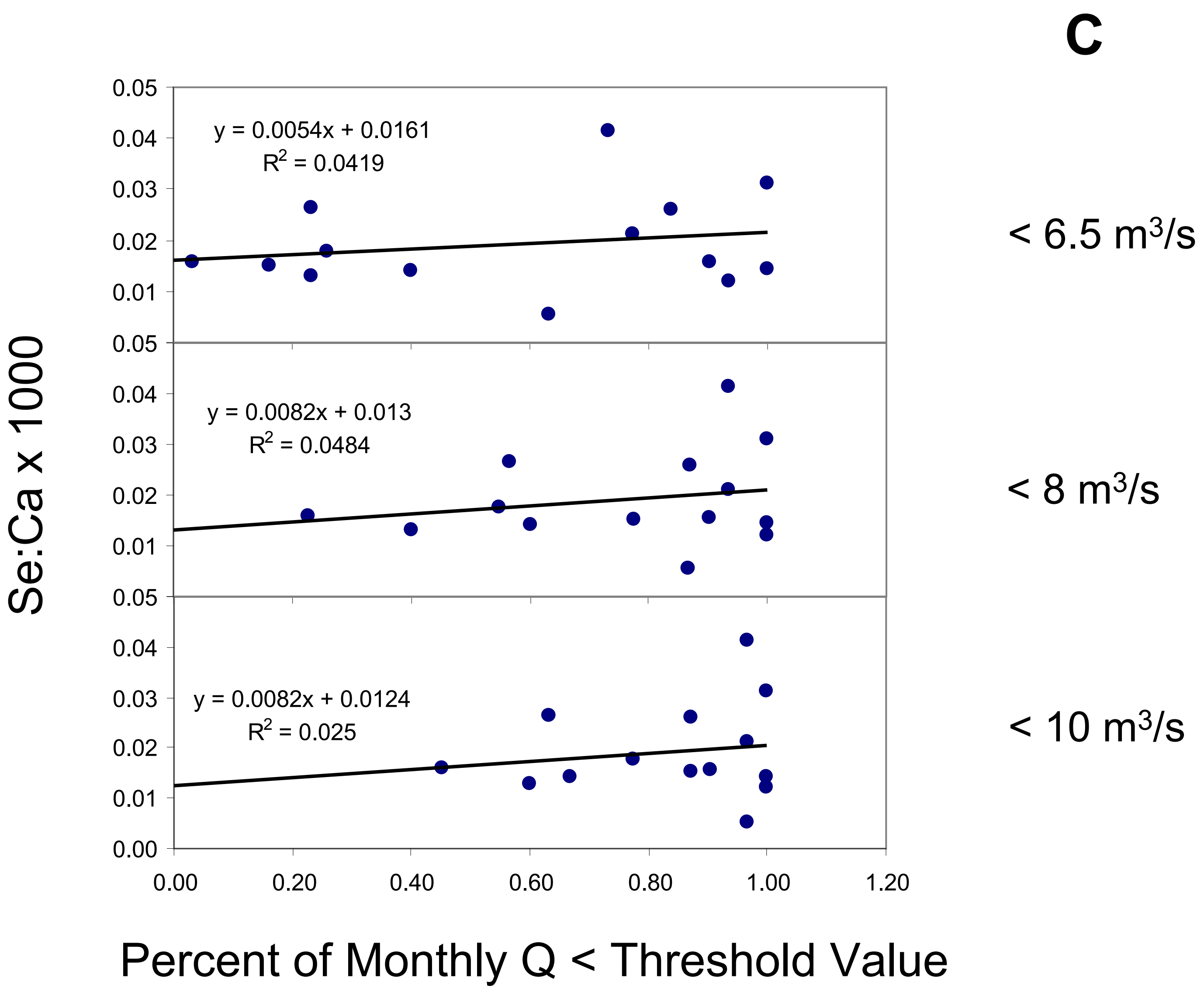

Figure S3, continued

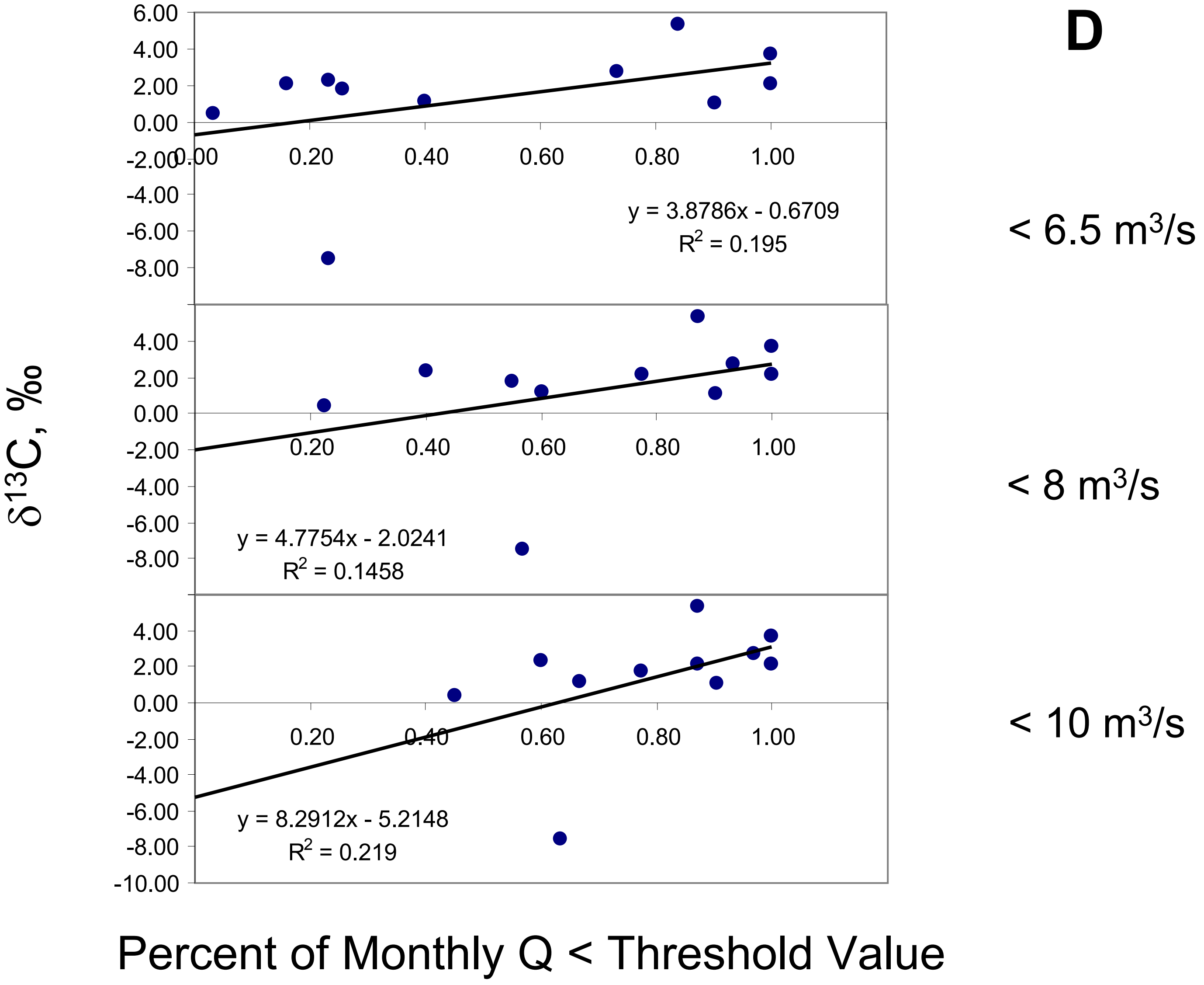

Figure S3, continued

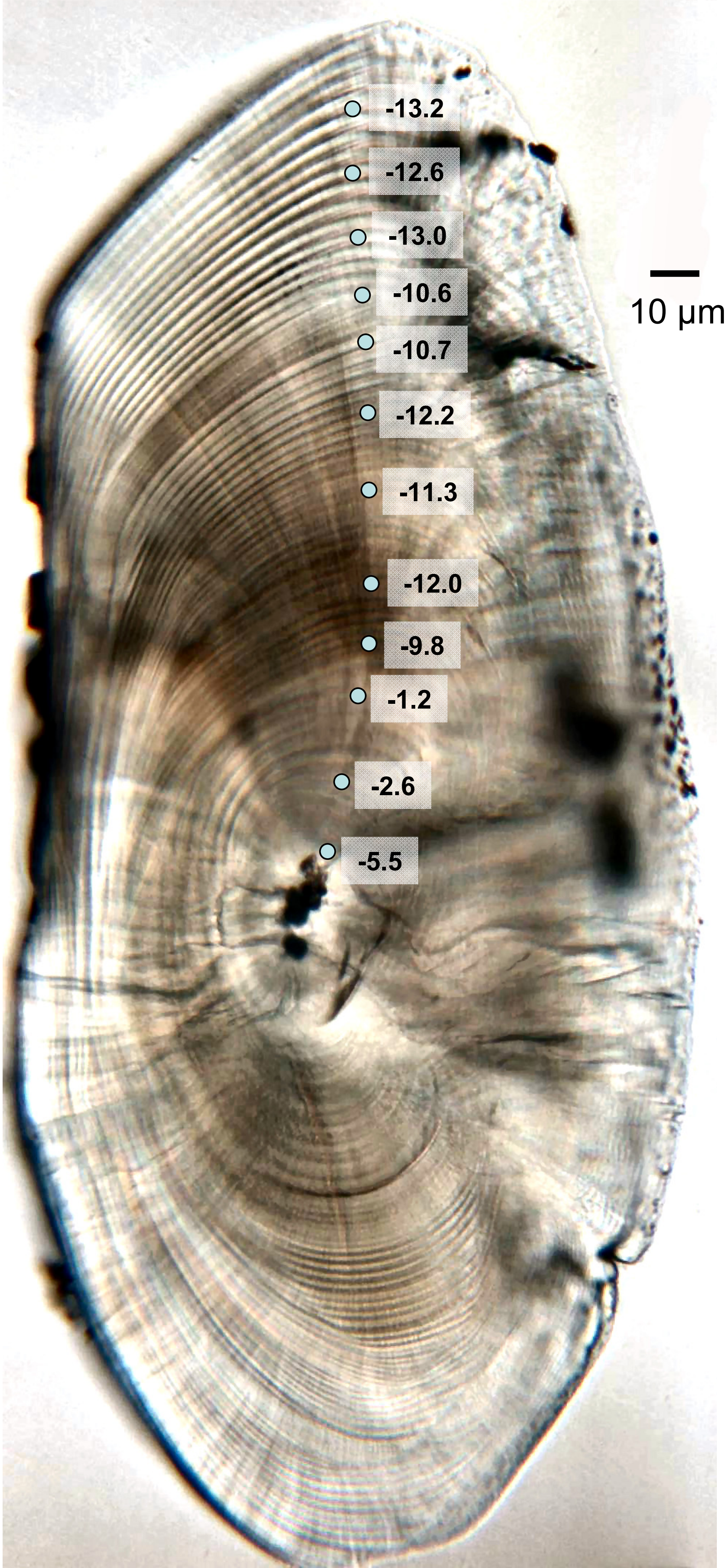

Figure S4.

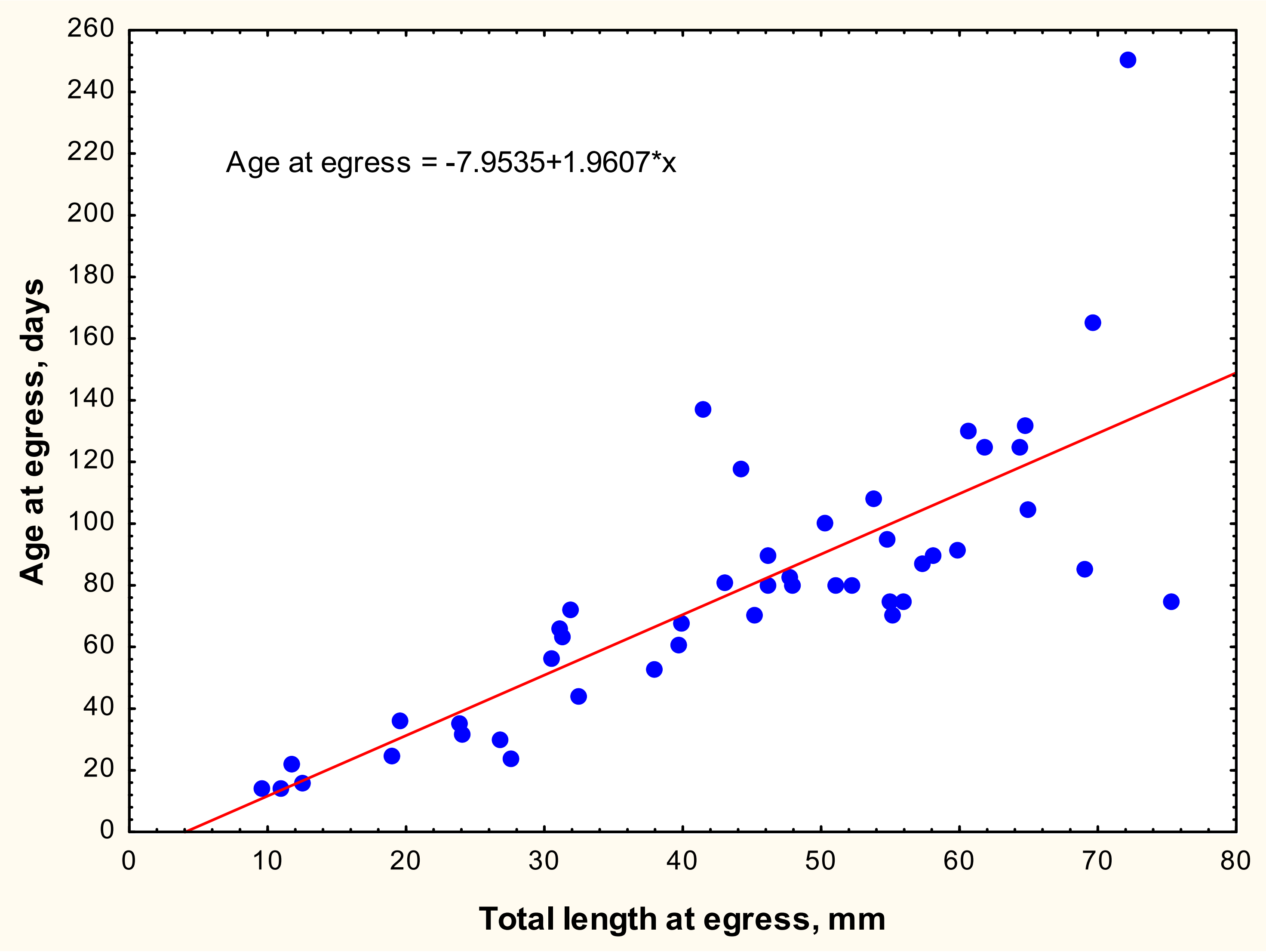
Age at egress = -7.9535+1.9607*x
Age at egress, days
Total length at egress, mm
0
20
40
60
80
100
120
140
160
180
200
220
240
260
0
10
20
30
40
50
60
70
80

Figure S5.

Supplemental tables.

Table S1. Carbon isotopic ratios ($\delta^{13}C$, ‰) and C concentrations (ppm) in dissolved inorganic carbon (DIC), dissolved organic carbon (DOC), and concentration-weighted average $\delta^{13}C$ at Boulder Camp, most downstream sampling site in the Little Colorado River, and the mainstem Colorado River upstream of the Little Colorado. Absolute difference in $\delta^{13}C$ between the two sites is computed for co-occurring dates.

Date	Site name	ppm DIC	DIC δ13C (PDB, ‰)	ppm DOC	DOC δ13C (PDB, ‰)	Fraction as DIC	Weighted Mean δ13C, ‰	Difference between Little Colorado River and MS, ‰
May-09	Boulder Camp	132.82	1.35	2.87	-2.37	0.979	1.28	9.47
Oct-09	Boulder Camp	78.58	3.69	3.38	-7.40	0.959	3.23	12.89
Jun-10	Boulder Camp	84.18	2.11	1.88	-7.42	0.978	1.90	
Jul-10	Boulder Camp	61.99	5.35	0.66	-11.33	0.989	5.18	10.47
Aug-10	Boulder Camp	77.38	0.42	3.08	-20.45	0.962	-0.38	8.78
Sep-10	Boulder Camp	70.94	1.15	3.22	-22.51	0.957	0.12	10.23
Oct-10	Boulder Camp	76.48	2.12	0.07	-23.54	0.999	2.09	10.53
Jul-11	Boulder Camp	69.42	1.05	0.30	-26.1	0.996	0.93	10.59
Aug-11	Boulder Camp	61.31	1.76	1.3	-27.0	0.979	1.14	10.77

Sep-11	Boulder Camp	26.27	-7.54	3.3	-26.3	0.889	-9.63	0.10
Oct-11	Boulder Camp	43.4	2.7					
					Mean	0.97	0.59	9.31
					S.E.	0.01	1.24	1.15
May-09	Mainstem	63.18	-7.48	3.96	-19.64	0.941	-8.20	
Oct-09	Mainstem	30.19	-7.92	3.92	-23.04	0.885	-9.66	
Jun-10	Mainstem							
Jul-10	Mainstem	23.94	-2.33	3.61	-24.93	0.869	-5.29	
Aug-10	Mainstem	31.16	-7.11	3.97	-25.23	0.887	-9.16	
Sep-10	Mainstem	34.17	-7.50	5.72	-25.66	0.857	-10.10	
Oct-10	Mainstem	31.12	-7.43	1.99	-24.17	0.940	-8.44	
Jul-11	Mainstem	23.35	-7.56	2.97	-26.1	0.887	-9.66	
Aug-11	Mainstem	27.68	-7.48	3.6	-26.3	0.886	-9.64	
Sep-11	Mainstem	27.47	-7.74	3.2	-26.6	0.895	-9.72	
Oct-11	Mainstem	24.0	-6.9					
					Mean	0.89	-8.87	
					S.E.	0.01	0.49	

Table S2. *In situ* carbon isotope analysis by SIMS

File	Sample	δ13C [‰ PDB]	± 2 SD	δ13C [measured]	2 SE	12C [10⁶ cps]	remarks
Analysis #		**[A]**	**[B]**	**[C]**	**[D]**	**[E]**	
	**(1) Sample change: Otolith # 2010 07 23 SN01**						
28	UWC-3			-39.5	0.77	7.2	EM HV adjust = 1852 V
29	UWC-3			-39.6	0.71	7.1	
30	UWC-3			-39.8	1.02	7.1	
31	UWC-3			-39.6	0.64	7.0	
32	20100723SN01-1	-5.5	0.9	-44.3	0.54	8.1	
33	20100723SN01-2	-2.6	0.9	-41.5	0.99	7.5	
34	20100723SN01-3	-1.2	0.9	-40.2	0.64	6.6	
35	20100723SN01-4	-12.0	0.9	-50.6	0.76	6.5	
36	20100723SN01-5	-12.2	0.9	-50.8	0.78	6.5	
37	20100723SN01-6	-11.3	0.9	-50.0	0.72	6.4	
38	20100723SN01-7	-12.2	0.9	-50.8	0.61	6.4	
39	UWC-3			-40.4	0.75	6.7	
40	UWC-3			-39.6	0.67	6.9	Cs reservoir = 142
41	UWC-3			-40.6	0.90	6.9	EM HV adjust; no change
42	UWC-3			-40.4	0.81	6.9	
	**Bracket (standard analyses 28-31, 39-42)**			**-39.9**			
	**±2 SD of bracketing standard analyses**			**0.9**			
43	20100723SN01-8	-10.3	1.6	-49.9	0.83	6.6	
44	20100723SN01-8	-10.7	1.6	-50.3	0.78	6.6	
45	20100723SN01-9	-10.6	1.6	-50.3	0.54	6.6	
46	20100723SN01-10	-13.0	1.6	-52.5	0.77	6.5	
47	20100723SN01-11	-12.6	1.6	-52.2	0.63	6.5	
48	20100723SN01-12	-13.2	1.6	-52.8	0.47	6.5	
49	20100723SN01-13	-9.8	1.6	-49.5	0.65	6.7	
50	20100723SN01-14	-1.7	1.6	-41.7	0.86	6.8	
51	20100723SN01-15	-0.8	1.6	-40.8	0.72	6.5	
52	20100723SN01-16	-12.2	1.6	-51.8	0.79	6.5	
53	20100723SN01-16	-10.6	1.6	-50.2	0.78	6.5	
54	UWC-3			-41.4	0.82	6.7	
55	UWC-3			-41.8	0.79	7.0	Cs reservoir = 143
56	UWC-3			-41.4	0.65	7.0	EM HV adjust; no change
57	UWC-3			-41.9	0.68	7.0	
	**Bracket (standard analyses 39-42, 54-57)**			**-40.9**			
	**±2 SD of bracketing standard analyses**			**1.6**			
	**(2) Sample change: Otolith # 20100710GCY01**						
58	UWC-3			-41.3	0.75	7.1	
59	UWC-3			-41.2	0.75	7.0	
60	UWC-3			-41.2	0.74	7.0	
61	UWC-3			-41.3	0.83	7.0	
62	20100710GCY01-1	-2.8	1.3	-43.6	1.02	7.2	
63	20100710GCY01-2	-2.5	1.3	-43.4	0.67	7.0	
64	20100710GCY01-3	-12.7	1.3	-53.1	0.60	7.1	
65	20100710GCY01-4	-11.5	1.3	-52.0	0.62	6.7	
66	20100710GCY01-5	-10.6	1.3	-51.1	1.00	6.8	
67	20100710GCY01-6	-12.1	1.3	-52.5	0.73	6.5	
68	20100710GCY01-7	-13.4	1.3	-53.9	0.97	6.5	
69	20100710GCY01-8	-12.0	1.3	-52.5	0.52	6.5	
70	UWC-3			-42.5	0.81	6.9	
71	UWC-3			-42.4	0.66	7.1	Cs reservoir = 143
72	UWC-3			-42.5	0.78	7.1	EM HV adjust = 1853 V
73	UWC-3			-42.5	0.70	7.1	
	**Bracket (standard analyses 58-61, 70-73)**			**-41.8**			
	**±2 SD of bracketing standard analyses**			**1.3**			
74	20100710GCY-9	-2.6	0.7	-44.2	0.73	6.9	
75	20100710GCY-10	-3.2	0.7	-44.8	0.84	6.9	
76	20100710GCY-11	-10.6	0.7	-51.9	0.78	6.9	
77	20100710GCY-12	-2.9	0.7	-44.5	0.67	6.8	
78	20100710GCY-12	-12.4	0.7	-53.6	0.83	7.3	
79	20100710GCY-13	-4.8	0.7	-46.4	0.60	7.5	
80	UWC-3			-43.2	0.81	7.1	
81	UWC-3			-42.2	0.68	7.1	EM HV adjust = 1855 V
82	UWC-3			-42.9	0.84	7.1	
83	UWC-3			-42.6	0.93	7.1	
	**Bracket (standard analyses 70-73, 80-83)**			**-42.6**			
	**±2 SD of bracketing standard analyses**			**0.7**			
	**(3) Sample change: Otolith # HBC-86**						
84	UWC-3			-42.8	0.79	7.0	
85	UWC-3			-42.9	0.85	7.0	

File	Sample	$\delta^{13}C$ [‰ PDB]	± 2 SD	$\delta^{13}C$ [measured]	2 SE	^{12}C [10^6 cps]	remarks
86	UWC-3			-43.0	1.10	7.0	
87	UWC-3			-43.2	0.65	7.0	
88	HBC-86-1	-1.0	0.8	-43.1	0.78	6.8	
89	HBC-86-2	-2.2	0.8	-44.3	0.71	6.5	
90	HBC-86-3	-2.2	0.8	-44.3	0.80	6.4	
91	HBC-86-4	-1.5	0.8	-43.6	0.67	6.4	
92	HBC-86-5	-3.3	0.8	-45.4	0.90	6.4	
93	HBC-86-6	-4.0	0.8	-46.0	0.86	6.4	
94	UWC-3			-43.7	0.87	6.8	
95	UWC-3			-43.6	1.04	7.0	Cs reservoir = 144
96	UWC-3			-42.4	0.73	7.2	EM HV adjust = 1858 V
97	UWC-3			~~-46.3~~	~~0.76~~	~~8.1~~	overlapping crack
98	UWC-3			-42.9	1.00	7.1	
	**Bracket (standard analyses 84-87, 94-98)**			**-43.1**			
	**±2 SD of bracketing standard analyses**			**0.8**			
	**(4) Sample change: Otolith # HBC-69**						
102	UWC-3			-43.4	1.00	7.3	
103	UWC-3			-43.4	0.65	7.2	
104	UWC-3			-43.5	0.77	7.2	EM HV adjust = 1860 V
105	UWC-3			-43.2	0.66	7.2	
106	HBC_69-1	0.0	0.3	-42.5	1.09	7.2	
107	HBC_69-2	-0.9	0.3	-43.5	1.00	6.9	
108	HBC_69-3	-2.2	0.3	-44.6	0.80	6.8	
109	HBC_69-4	-3.7	0.3	-46.1	0.93	6.8	
110	HBC_69-5	-3.1	0.3	-45.5	0.63	6.7	
111	HBC_69-6	-10.8	0.3	-52.9	0.60	6.8	
112	HBC_69-6	-11.3	0.3	-53.3	0.82	6.8	
113	HBC_69-8	-5.6	0.3	-47.9	0.88	6.7	
114	UWC-3			-43.3	0.84	7.0	
115	UWC-3			-43.4	0.71	7.2	Cs reservoir = 145
116	UWC-3			-43.8	0.71	7.3	EM HV adjust = 1863 V
117	UWC-3			-43.4	0.73	7.3	
	**Bracket (standard analyses 102-105, 114-117)**			**-43.4**			
	**±2 SD of bracketing standard analyses**			**0.3**			
	**(5) Sample change: Otolith # HBC-58**						
118	UWC-3			-43.1	0.81	7.2	
119	UWC-3			-43.1	0.69	7.1	
120	UWC-3			-43.3	0.70	7.2	
121	UWC-3			-42.7	0.94	7.1	
122	HBC_58-1	-0.6	0.8	-42.8	0.72	6.9	
123	HBC_58-2	-3.3	0.8	-45.4	0.90	6.8	
124	HBC_58-3	-2.6	0.8	-44.7	0.82	6.9	
125	HBC_58-4	-3.4	0.8	-45.5	0.62	6.8	
126	HBC_58-5	-4.5	0.8	-46.5	0.93	6.8	
127	HBC_58-6	-3.8	0.8	-45.9	0.63	6.8	
128	HBC_58-7	-5.1	0.8	-47.1	0.49	6.7	
129	HBC_58-8	-4.0	0.8	-46.1	0.87	6.7	
130	UWC-3			-43.9	0.86	7.1	
131	UWC-3			-42.9	0.65	7.0	EM HV adjust = 1867 V
132	UWC-3			-42.7	0.72	6.9	
133	UWC-3			-43.3	0.84	7.0	
	**Bracket (standard analyses 118-121, 130-133)**			**-43.1**			
	**±2 SD of bracketing standard analyses**			**0.8**			
	**(6) Sample change: Otolith # HBC-89**						
135	UWC-3			-43.1	0.85	7.4	
136	UWC-3			-42.9	0.90	7.4	EM HV adjust = 1869 V
137	UWC-3			-43.1	0.91	7.5	
138	UWC-3			-43.3	0.71	7.4	
139	HBC_89-1	-3.7	0.95	-45.9	0.77	8.0	
140	HBC_89-2	-1.1	0.95	-43.4	0.64	7.1	
141	HBC_89-3	-4.3	0.95	-46.5	0.65	7.9	
142	HBC_89-4	-2.6	0.95	-44.8	0.60	7.1	
143	HBC_89-5	-3.7	0.95	-45.9	0.75	7.2	
144	HBC_89-6	-3.5	0.95	-45.7	0.60	7.1	
145	HBC_89-7	-4.1	0.95	-46.3	0.72	7.1	
146	HBC_89-8			~~-46.2~~	~~0.86~~	~~8.4~~	high count rate - organics?
147	HBC_89-9	-2.2	0.95	-44.5	0.70	7.0	
149	UWC-3			-44.4	0.82	7.2	
150	UWC-3			-42.9	0.80	7.2	EM HV adjust = 1873 V
151	UWC-3			-43.3	0.91	7.2	

File	Sample	$\delta^{13}C$ [‰ PDB]	± 2 SD	$\delta^{13}C$ [measured]	2 SE	^{12}C [10^6 cps]	remarks
152	UWC-3			-43.0	0.81	7.2	
	**Bracket (standard analyses 135-138, 149-152)**			**-43.2**			
	**±2 SD of bracketing standard analyses**			**1.0**			
	**(7) Sample change: Otolith # 20101020EF02**						
154	UWC-3			-42.6	0.78	7.9	
155	UWC-3			-42.5	0.54	7.8	EM HV adjust = 1879 V
156	UWC-3			-42.8	0.96	7.7	
157	UWC-3			-42.4	0.58	7.7	
158	20101020EF02-1	-2.2	0.46	-43.9	0.91	7.5	
159	20101020EF02-2	-2.5	0.46	-44.2	0.56	7.3	
160	20101020EF02-3	-4.3	0.46	-45.9	0.49	7.3	
161	20101020EF02-4	-11.4	0.46	-52.7	0.64	7.3	
162	20101020EF02-5	-12.2	0.46	-53.5	0.73	7.3	
163	20101020EF02-6	-9.6	0.46	-51.0	0.89	7.4	
164	UWC-3			-42.6	0.99	7.5	
165	UWC-3			-42.8	0.81	7.6	
166	UWC-3			-42.6	0.72	7.6	EM HV adjust; no change
167	UWC-3			-43.1	0.82	7.5	
	**Bracket (standard analyses 154-157, 164-167)**			**-42.7**			
	**±2 SD of bracketing standard analyses**			**0.5**			
168	20101020EF02-7	-10.2	0.63	-51.8	0.60	7.3	
169	20101020EF02-8	-11.3	0.63	-52.9	0.49	7.2	
170	20101020EF02-9	-10.9	0.63	-52.5	0.65	7.2	
171	20101020EF02-10	-11.2	0.63	-52.8	0.68	7.0	
172	20101020EF02-11	-12.3	0.63	-53.8	0.73	7.1	
173	20101020EF02-12	-11.8	0.63	-53.3	0.78	7.1	
174	20101020EF02-13	-11.4	0.63	-52.9	0.65	7.1	
175	UWC-3			-42.9	0.67	7.4	
176	UWC-3			-43.1	0.69	7.3	
177	UWC-3			-42.8	0.94	7.4	EM HV adjust; no change
178	UWC-3			-43.5	0.65	7.3	
	**Bracket (standard analyses 164-167, 175-178)**			**-42.9**			
	**±2 SD of bracketing standard analyses**			**0.6**			
179	20101020EF02-14	-10.9	0.82	-52.9	0.82	7.1	
180	20101020EF02-15	-11.8	0.82	-53.7	0.90	7.1	
181	20101020EF02-16	-4.4	0.82	-46.6	0.45	7.0	
182	20101020EF02-17	-11.7	0.82	-53.6	0.79	7.1	
183	20101020EF02-18	-4.4	0.82	-46.7	0.97	7.5	
184	20101020EF02-19	-3.9	0.82	-46.2	0.64	7.1	
185	20101020EF02-20	-6.1	0.82	-48.3	0.90	7.0	
186	20101020EF02-21	-6.2	0.82	-48.3	0.66	6.9	
187	UWC-3			-44.0	0.85	7.2	
188	UWC-3			-43.5	0.86	7.2	EM HV adjust = 1881 V
189	UWC-3			-43.4	0.73	7.2	
190	UWC-3			-42.9	0.58	7.2	
	**Bracket (standard analyses 175-178, 187-190)**			**-43.3**			
	**±2 SD of bracketing standard analyses**			**0.8**			
	**(8) Sample change: Otolith # HBC-61**						
191	UWC-3			-43.4	0.77	7.0	
192	UWC-3			-43.4	0.68	7.3	Cs reservoir = 148
193	UWC-3			-42.8	0.69	7.4	EM HV adjust = 1882 V
194	UWC-3			-42.8	0.88	7.5	
195	HBC-61	-1.6	0.85	-43.9	0.65	7.3	
196	HBC-61-2	-1.5	0.85	-43.9	0.97	7.1	
197	HBC-61-3	-3.1	0.85	-45.4	0.84	7.1	
198	HBC-61-3	-3.9	0.85	-46.1	0.64	7.1	
199	HBC-61-4	-2.6	0.85	-45.0	0.72	7.1	
200	HBC-61-5	-5.6	0.85	-47.8	0.79	7.1	
201	HBC-61-6	-3.4	0.85	-45.7	0.64	7.0	
202	HBC-61-7	-2.8	0.85	-45.1	0.95	7.2	
203	UWC-3			-44.1	0.97	7.3	
204	UWC-3			-43.0	0.56	7.4	EM HV adjust = 1884 V
205	UWC-3			-43.4	0.91	7.3	
206	UWC-3			-43.5	0.59	7.3	
	**Bracket (standard analyses 191-194, 203-206)**			**-43.3**			
	**±2 SD of bracketing standard analyses**			**0.9**			
207	HBC_61-9	-1.6	0.75	-44.2	0.62	6.9	
208	HBC_61-10	-1.7	0.75	-44.3	0.64	6.9	
209	HBC_61-11	-2.6	0.75	-45.2	0.86	6.9	
210	HBC_61-12	-2.5	0.75	-45.1	0.45	6.9	

File	Sample	$\delta^{13}C$ [‰ PDB]	± 2 SD	$\delta^{13}C$ [measured]	2 SE	^{12}C [10^6 cps]	remarks
211	HBC_61-13	-3.1	0.75	-45.7	0.56	6.9	
212	HBC_61-14	-5.5	0.75	-48.0	0.61	6.9	
213	HBC_61-15	-7.3	0.75	-49.7	0.86	7.0	
214	HBC_61-16	-2.4	0.75	-45.1	0.70	6.8	
						0.0	
215	UWC-3			-44.1	0.71	7.1	
216	UWC-3			-43.4	0.76	7.0	EM HV adjust = 1887 V
217	UWC-3			-43.5	0.70	7.1	
218	UWC-3			-43.7	0.63	7.1	
	**Bracket (standard analyses 203-206, 215-218)**			**-43.6**			
	**±2 SD of bracketing standard analyses**			**0.7**			
	**(9) Sample change: Otolith # 201022 HP02**						
220	UWC-3			-43.5	0.81	7.2	
221	UWC-3			-42.6	0.78	7.2	Cs reservoir = 149
222	UWC-3			-43.2	0.69	7.2	EM HV adjust = 1890 V
223	UWC-3			-43.2	0.70	7.2	
224	20101022HP02			~~-46.5~~	~~0.78~~	~~8.0~~	high count rate - organics?
225	20101022HP02-2	-2.2	0.7	-44.5	0.94	7.3	
226	20101022HP02-3	-2.8	0.7	-45.1	0.68	7.0	
227	20101022HP02-4	-3.9	0.7	-46.2	0.85	6.9	
228	20101022HP02-5	-3.5	0.7	-45.8	0.82	6.8	
229	20101022HP02-6	-3.7	0.7	-46.0	0.80	6.8	
230	20101022HP02-7	-3.6	0.7	-45.9	0.63	6.7	
231	20101022HP02-8	-2.9	0.7	-45.2	0.90	6.7	
232	UWC-3			-43.8	0.67	7.1	
233	UWC-3			-43.4	0.89	7.0	EM HV adjust = 1891 V
234	UWC-3			-43.5	0.65	7.2	Cs reservoir = 150
235	UWC-3			-43.4	0.71	7.4	
	**Bracket (standard analyses 220-223, 232-235)**			**-43.3**			
	**±2 SD of bracketing standard analyses**			**0.7**			
236	20101022HP02-1	-4.5	0.8	-46.9	0.58	7.3	
237	20101022HP02-2	-3.4	0.8	-45.8	0.87	7.0	
238	20101022HP02-3	-2.3	0.8	-44.8	0.64	7.0	
239	20101022HP02-4	-10.0	0.8	-52.1	0.77	6.9	
240	20101022HP02-5	-11.1	0.8	-53.2	0.56	6.9	
241	20101022HP02-6	-3.1	0.8	-45.5	0.73	6.9	
242	20101022HP02-7	-5.4	0.8	-47.7	0.81	7.0	
243	20101022HP02-8	-4.2	0.8	-46.5	0.79	7.0	
244	UWC-3			-44.1	0.77	7.2	
245	UWC-3			-43.4	0.68	7.2	EM HV adjust = 1895 V
246	UWC-3			-42.8	0.74	7.2	
247	UWC-3			-42.9	0.73	7.2	
	**Bracket (standard analyses 232-235, 244-247)**			**-43.4**			
	**±2 SD of bracketing standard analyses**			**0.8**			
	**(10) Sample change: Otolith # 20101026 EF04**						
248	UWC-3			-43.8	0.82	7.3	
249	UWC-3			-43.7	0.71	7.3	
250	UWC-3			-43.1	0.83	7.2	EM HV adjust = 1897 V
251	UWC-3			-42.9	0.61	7.3	
252	20101026EF04-1	0.6	0.7	-42.0	0.69	7.0	
253	20101026EF04-2	-3.4	0.7	-45.8	0.82	7.8	
254	20101026EF04-3	-1.4	0.7	-43.9	0.60	6.9	
255	20101026EF04-3	-3.6	0.7	-46.0	0.87	6.8	
256	20101026EF04-4	-3.2	0.7	-45.6	0.70	6.8	
257	20101026EF04-5	-2.5	0.7	-45.0	0.78	6.8	
258	20101026EF04-6	-3.4	0.7	-45.9	0.54	6.8	
259	20101026EF04-7	-5.1	0.7	-47.4	0.70	7.1	
260	UWC-3			-43.7	0.81	7.0	
261	UWC-3			-43.2	0.52	7.0	EM HV adjust = 1899 V
262	UWC-3			-43.8	0.72	7.2	Cs reservoir = 151
263	UWC-3			-43.4	0.79	7.3	
	**Bracket (standard analyses 248-251, 260-263)**			**-43.5**			
	**±2 SD of bracketing standard analyses**			**0.7**			
264	20101026EF04-9	-1.6	0.7	-44.4	0.70	7.0	
265	20101026EF04-10	-0.7	0.7	-43.6	0.76	7.0	
266	20101026EF04-11	-11.3	0.7	-53.7	0.55	7.0	
267	20101026EF04-12	-11.4	0.7	-53.8	0.85	7.0	
268	20101026EF04-13	-10.3	0.7	-52.7	0.72	6.9	
269	20101026EF04-14	-9.6	0.7	-52.1	0.69	6.9	
270	20101026EF04-15	-5.1	0.7	-47.8	0.72	6.9	
271	20101026EF04-16	-3.0	0.7	-45.7	0.84	6.8	

File	Sample	$\delta^{13}C$ [‰ PDB]	± 2 SD	$\delta^{13}C$ [measured]	2 SE	^{12}C [10^6 cps]	remarks
272	UWC-3			-44.3	0.69	7.2	
273	UWC-3			-43.8	0.81	7.2	EM HV adjust = 1901 V
274	UWC-3			-43.7	0.73	7.2	
275	UWC-3			-44.1	0.90	7.2	
	**Bracket (standard analyses 260-263, 272-275)**			**-43.8**			
	**±2 SD of bracketing standard analyses**			**0.7**			
	**(11) Sample change: Otolith # 201024 HP05**						
278	UWC-3			-43.3	0.76	7.1	
279	UWC-3			-43.4	0.61	7.1	EM HV adjust = 1908 V
280	UWC-3			-42.8	0.64	7.1	
281	UWC-3			-42.7	0.59	7.0	
282	20101024HP05-1	-0.8	0.6	-42.9	0.65	6.9	
283	20101024HP05-2	-1.8	0.6	-43.9	0.83	6.5	
284	20101024HP05-3	-3.7	0.6	-45.7	0.66	6.5	
285	20101024HP05-4	-4.3	0.6	-46.2	0.85	6.5	
286	20101024HP05-5	-4.5	0.6	-46.4	0.71	6.5	
287	20101024HP05-6	-11.5	0.6	-53.1	0.96	6.4	
288	20101024HP05-7	-5.3	0.6	-47.2	0.94	6.4	
289	20101024HP05-8	-11.5	0.6	-53.2	0.98	6.4	
290	UWC-3			-42.7	0.91	6.7	
291	UWC-3			-43.1	0.90	6.9	Cs reservoir = 153
292	UWC-3			-42.6	0.91	7.0	
293	UWC-3			-43.3	0.93	7.0	
	**Bracket (standard analyses 278-281, 290-293)**			**-43.0**			
	**±2 SD of bracketing standard analyses**			**0.6**			
294	20101024HP04-9	-11.3	0.7	-53.1	0.82	6.7	
295	20101024HP04-10	-13.0	0.7	-54.7	0.51	6.6	
296	20101024HP04-11	-12.1	0.7	-53.8	0.97	6.6	
297	20101024HP04-12	-12.2	0.7	-53.9	0.81	6.5	
298	20101024HP04-13	-13.0	0.7	-54.7	0.58	6.4	
299	20101024HP04-14	-12.9	0.7	-54.5	0.83	6.6	
300	20101024HP04-15	-12.8	0.7	-54.5	0.65	6.5	
301	20101024HP04-16	-12.3	0.7	-54.0	0.66	6.4	
302	UWC-3			-43.3	0.72	6.8	
303	UWC-3			-42.9	0.69	6.7	EM HV adjust = 1909 V
304	UWC-3			-43.5	0.78	6.9	Cs reservoir = 154
305	UWC-3			-43.4	0.94	7.0	
	**Bracket (standard analyses 290-293, 302-305)**			**-43.1**			
	**±2 SD of bracketing standard analyses**			**0.7**			
	**(12) Sample change: Otolith # 201022HP05**						
306	UWC-3			-43.4	0.62	6.9	
308	UWC-3			-43.8	0.86	7.2	
309	UWC-3			-43.5	0.68	7.2	EM HV adjust; no change
310	UWC-3			-44.4	0.81	7.2	
311	20101022HP05	-2.7	0.8	-45.6	0.58	6.8	
312	20101022HP05-2	-1.2	0.8	-44.2	0.56	6.8	
313	20101022HP05-3	-1.5	0.8	-44.5	0.74	6.7	
314	20101022HP05-4	-2.6	0.8	-45.5	0.80	6.7	
315	20101022HP05-5	-4.7	0.8	-47.5	0.80	6.7	
316	20101022HP05-6	-4.9	0.8	-47.8	0.64	6.7	
317	20101022HP05-7	-5.7	0.8	-48.6	0.94	6.6	
318	20101022HP05-8	-4.2	0.8	-47.1	0.78	6.7	
319	UWC-3			-44.2	0.92	7.0	
320	UWC-3			-44.5	0.71	7.2	Cs reservoir = 155
321	UWC-3			-44.0	0.74	7.2	EM HV adjust = 1910 V
322	UWC-3			-43.7	0.69	7.3	
	**Bracket (standard analyses 306-310, 319-322)**			**-44.0**			
	**±2 SD of bracketing standard analyses**			**0.8**			
323	20101022HP05-1	-3.7	0.7	-46.7	0.85	6.9	
324	20101022HP05-2	-5.5	0.7	-48.4	0.75	6.9	
325	20101022HP05-3	-10.8	0.7	-53.4	0.72	6.9	
326	20101022HP05-4	-12.3	0.7	-54.9	0.82	6.9	
327	20101022HP05-5	-11.0	0.7	-53.7	0.74	6.9	
328	20101022HP05-6	-10.6	0.7	-53.3	0.85	7.0	
329	20101022HP05-7	-11.5	0.7	-54.1	0.62	6.8	
330	20101022HP05-8	-12.6	0.7	-55.2	0.67	6.9	
331	UWC-3			-44.4	0.81	7.2	
332	UWC-3			-43.6	0.68	7.2	EM HV adjust = 1913 V
333	UWC-3			-43.9	0.62	7.2	
334	UWC-3			-43.7	0.77	7.3	
	**Bracket (standard analyses 319-322, 331-334)**			**-44.0**	**0.77**		

File	Sample	$\delta^{13}C$ [‰ PDB]	± 2 SD	$\delta^{13}C$ [measured]	2 SE	^{12}C [10^6 cps]	remarks
	**±2 SD of bracketing standard analyses**			**0.7**			
	**(13) Sample change: Otolith # HBC-95**						
336	UWC-3			-42.6	0.81	6.9	
337	UWC-3			-43.4	0.71	6.9	
338	UWC-3			-42.9	0.73	6.8	
339	UWC-3			~~-45.9~~	~~0.79~~	~~7.8~~	high yield, crosscutting vein
340	UWC-3			~~-45.6~~	~~0.83~~	~~7.8~~	high yield, crosscutting vein
341	UWC-3			-43.3	0.80	7.1	
342	UWC-3			-43.5	0.89	7.1	
343	HBC_95-1	-1.1	0.6	-43.5	0.71	7.0	
344	HBC_95-2	-1.7	0.6	-44.0	0.69	6.9	
345	HBC_95-3	-1.0	0.6	-43.3	0.82	6.8	
346	HBC_95-4	-1.4	0.6	-43.7	0.67	6.7	
347	HBC_95-5	-2.4	0.6	-44.7	0.80	6.8	
348	HBC_95-6	-2.2	0.6	-44.5	0.84	6.7	
349	HBC_95-7	-3.8	0.6	-46.1	0.74	6.7	
350	HBC_95-8	-2.2	0.6	-44.5	0.63	6.7	
351	UWC-3			-43.8	0.76	7.0	
352	UWC-3			-43.1	0.72	6.9	EM HV adjust = 1917 V
353	UWC-3			-42.9	0.68	6.9	
354	UWC-3			-43.0	0.92	6.8	
	**Bracket (standard analyses 336-342, 351-354)**			**-43.2**			
	**±2 SD of bracketing standard analyses**			**0.6**			
	**(14) Sample change: Otolith # 20101016 HP02**						
355	UWC-3			-44.2	0.64	7.1	
356	UWC-3			-44.8	0.65	7.2	
357	UWC-3			-43.8	0.69	7.2	EM HV adjust = 1920 V
358	UWC-3			-43.7	0.61	7.1	
359	20101016HP02-1			~~-46.9~~	~~0.77~~	~~8.5~~	high count rate - organics?
360	20101016HP02-2	-2.2	0.9	-45.3	0.84	6.8	
361	20101016HP02-3	-4.0	0.9	-47.0	0.83	6.7	
362	20101016HP02-4	-5.1	0.9	-48.1	0.64	7.1	
363	20101016HP02-5	-5.5	0.9	-48.5	0.63	7.2	
364	20101016HP02-6	-3.8	0.9	-46.8	0.98	6.6	
365	20101016HP02-7	-3.8	0.9	-46.9	0.87	6.5	
366	20101016HP02-8	-2.9	0.9	-46.0	0.65	6.5	
367	UWC-3			-43.8	0.83	6.7	
368	UWC-3			-44.6	0.89	6.6	EM HV adjust = 1922 V
369	UWC-3			-44.1	0.83	6.9	Cs reservoir = 157
370	UWC-3			-43.6	0.89	7.0	
	**Bracket (standard analyses 355-358, 367-370)**			**-44.1**			
	**±2 SD of bracketing standard analyses**			**0.9**			
371	20101016HP02-9	-2.2	0.8	-45.3	0.51	6.8	
372	20101016HP02-10	-9.6	0.8	-52.4	0.98	6.7	
373	20101016HP02-11	-8.2	0.8	-51.1	0.73	6.7	
374	20101016HP02-12	-2.1	0.8	-45.2	0.71	6.6	
375	20101016HP02-13	-11.1	0.8	-53.8	0.91	6.6	
376	20101016HP02-14			~~-50.1~~	~~0.64~~	~~7.3~~	high count rate - organics?
377	20101016HP02-15			~~-50.5~~	~~0.74~~	~~7.5~~	high count rate - organics?
378	20101016HP02-16	-4.9	0.8	-47.9	0.73	6.6	
379	20101016HP02-17	-5.5	0.8	-48.4	0.49	6.6	
380	UWC-3			-44.7	0.60	6.7	
391	UWC-3			~~-46.4~~	~~0.58~~	~~6.3~~	EM HV adjust = 1926 V, pit placed in vein
382	UWC-3			-43.6	0.72	6.7	
383	UWC-3			-43.8	0.66	6.7	
384	UWC-3			-44.0	0.67	6.6	
	**Bracket (standard analyses 367-370, 380-384)**			**-44.0**			
	**±2 SD of bracketing standard analyses**			**0.8**			
	**(15) Sample change: Otolith # HPC-77**						
385	UWC-3			-43.7	0.60	6.7	
386	UWC-3			-43.9	0.62	6.9	Cs reservoir = 158
387	UWC-3			-44.6	0.68	6.9	EM HV adjust = 1928 V
388	UWC-3			-43.3	0.93	6.9	
389	HBC-77-1	-1.3	0.8	-44.3	0.77	7.0	
390	HBC-77-2	-1.0	0.8	-44.0	0.66	6.6	
391	HBC-77-3	-1.2	0.8	-44.2	0.67	6.5	
392	HBC-77-4	-1.9	0.8	-44.8	0.77	6.6	
393	HBC-77-5	-3.0	0.8	-45.9	0.64	6.6	
394	HBC-77-6	-1.8	0.8	-44.8	0.86	6.6	
395	HBC-77-7	-2.6	0.8	-45.6	0.76	6.6	
396	HBC-77-8	-2.5	0.8	-45.4	0.72	6.6	

File	Sample	$\delta^{13}C$ [‰ PDB]	± 2 SD	$\delta^{13}C$ [measured]	2 SE	^{12}C [10^6 cps]	remarks
397	HBC-77-9	-2.8	0.8	-45.7	0.71	6.6	
398	UWC-3			-43.6	0.63	6.8	
399	UWC-3			-44.3	0.71	6.8	
400	UWC-3			-44.0	0.75	6.8	EM HV adjust = 1930 V
401	UWC-3			-43.9	0.76	6.8	
	**Bracket (standard analyses 385-388, 398-401)**			**-43.9**			
	**±2 SD of bracketing standard analyses**			**0.8**			
	**(16) Sample change: Otolith # HBC-94**						
402	UWC-3			-43.7	0.78	7.1	
403	UWC-3			-44.2	0.63	7.2	Cs reservoir = 159
404	UWC-3			-44.4	0.73	7.2	
405	UWC-3			-43.9	0.85	7.3	
406	HBC_94-1	-2.5	0.6	-45.7	0.80	7.0	
407	HBC_94-2	-0.4	0.6	-43.7	0.64	7.0	
408	HBC_94-3	-0.5	0.6	-43.7	0.77	7.0	
409	HBC_94-4	-0.9	0.6	-44.2	0.82	6.9	
410	HBC_94-5	-1.0	0.6	-44.2	0.69	6.9	
411	HBC_94-6	-1.7	0.6	-44.9	0.64	6.9	
412	HBC_94-7	-2.8	0.6	-45.9	0.59	6.8	
413	HBC_94-8	-2.1	0.6	-45.3	0.94	7.0	
414	UWC-3			-44.6	0.66	7.3	
415	UWC-3			-44.2	0.86	7.3	
416	UWC-3			-43.8	0.72	7.3	
417	UWC-3			-44.3	0.56	7.3	
	**Bracket (standard analyses 402-405, 414-417)**			**-44.1**			
	**±2 SD of bracketing standard analyses**			**0.6**			

Column [A]: Carbon isotope ratios in PDB scale, corrected for instrumental bias. The UWC-3 calcite standard has a calibrated $\delta^{13}C$ of -0.91‰ PDB [Kozdon et al., 2009]

Column [B]: ±2 SD of the bracketing standard analyses

Column [C]: Raw measured $^{13}C/^{12}C$ ratios

Column [D]: Internal error of a single analysis calculated as the twice of the standard error of the mean of 20 cycles

Column [E]: Secondary intensity of $^{12}C^-$ ions in counts per second (cps)

Reference:

Kozdon, R., T. Ushikubo, N. T. Kita, M. Spicuzza, and J. W. Valley (2009). Intratest oxygen isotope variability in the planktonic foraminifer *N. pachyderma* : Real vs. apparent vital effects by ion microprobe, Chem. Geol. 258, 327-337.

Table S3. Mean (± 95% confidence intervals) monthly temperatures, 2008-2012, in the Little Colorado River (LCR) and the Colorado River mainstem (Mainstem), 2008-2012. Between-river differences are given as ΔT. Data are from USGS gaging stations 09402300 (LCR) and 09402500 (Mainstem).

Date	LCR mean	LCR 95% c.i.	Mainstem mean	Mainstem 95% c.i.	ΔT
Jan-08	11.0	1.07	8.7	0.19	2.3
Feb-08	8.3	0.98	8.1	0.11	0.3
Mar-08	11.8	0.81	8.9	0.21	3.0
Apr-08	16.5	0.65	9.5	0.12	7.0
May-08	19.8	0.54	10.3	0.16	9.5
Jun-08	22.3	0.40	11.8	0.23	10.6
Jul-08	23.8	0.26	13.0	0.10	10.8
Aug-08	23.2	0.19	13.3	0.08	9.9
Sep-08	21.1	0.18	13.6	0.06	7.5
Oct-08	18.1	0.47	13.2	0.19	4.9
Nov-08	16.2	0.32	12.7	0.18	3.5
Dec-08	14.2	0.40	11.1	0.36	3.1
Jan-09	14.5	0.42	9.4	0.14	5.0
Feb-09	14.2	0.60	8.9	0.12	5.3
Mar-09	13.0	0.66	9.4	0.16	3.6
Apr-09	17.7	0.62	10.3	0.23	7.4
May-09	21.2	0.29	11.9	0.14	9.3
Jun-09	21.7	0.42	12.3	0.12	9.4
Jul-09	23.4	0.22	12.8	0.09	10.6
Aug-09	22.6	0.21	12.7	0.07	9.9
Sep-09	21.2	0.41	12.9	0.09	8.4
Oct-09	17.7	0.52	11.7	0.17	6.0
Nov-09	16.0	0.43	11.4	0.21	4.6
Dec-09	14.1	0.35	10.5	0.20	3.6
Jan-10	13.0	0.95	9.0	0.14	4.1
Feb-10	14.6	0.79	8.6	0.08	6.0
Mar-10	13.5	0.80	9.0	0.11	4.6
Apr-10	14.5	0.65	9.7	0.17	4.8
May-10	19.1	0.61	10.7	0.24	8.4
Jun-10	22.3	0.41	12.1	0.19	10.2
Jul-10	23.8	0.27	12.3	0.07	11.4
Aug-10	23.5	0.35	12.3	0.09	11.2
Sep-10	21.0	0.29	12.8	0.11	8.2
Oct-10	18.6	0.51	11.9	0.20	6.7
Nov-10	15.5	0.62	10.8	0.21	4.7
Dec-10	15.3	0.41	10.7	0.19	4.6
Jan-11	14.1	0.40	9.0	0.13	5.1
Feb-11	14.3	0.57	8.3	0.11	6.0
Mar-11	15.2	0.46	8.8	0.10	6.4
Apr-11	17.8	0.47	9.5	0.15	8.3
May-11	19.8	0.47	10.1	0.15	9.7
Jun-11	22.0	0.35	11.4	0.23	10.6
Jul-11	23.4	0.20	13.5	0.22	9.9

Aug-11	23.5	0.18	14.6	0.08	8.9
Sep-11	20.9	0.40	15.0	0.07	5.9
Oct-11	18.3	0.39	14.7	0.13	3.6
Nov-11	15.5	0.34	14.1	0.15	1.4
Dec-11	13.4	0.66	12.3	0.29	1.0
Jan-12	14.6	0.22	10.0	0.19	4.6
Feb-12	15.6	0.21	9.4	0.08	6.2
Mar-12	16.9	0.48	9.6	0.17	7.3
Apr-12	18.7	0.71	10.5	0.22	8.2
May-12	21.0	0.32	11.8	0.12	9.2
Jun-12	22.8	0.24	12.3	0.10	10.5
Jul-12	23.4	0.20	12.0	0.10	11.4
Aug-12	23.5	0.17	12.0	0.06	11.5
Sep-12	21.5	0.31	12.4	0.08	9.1
Oct-12	18.7	0.51	11.3	0.20	7.4
Nov-12	16.1	0.39	10.1	0.19	6.0
Dec-12	14.6	0.47	9.7	0.20	4.9
